# Suppression of Immunodominant Antitumor and Antiviral CD8^+^ T Cell Responses by Indoleamine 2,3-Dioxygenase

**DOI:** 10.1371/journal.pone.0090439

**Published:** 2014-02-28

**Authors:** Mateusz Rytelewski, Courtney E. Meilleur, Maryam Atef Yekta, Peter A. Szabo, Nitan Garg, Todd D. Schell, Anthony M. Jevnikar, Shayan Sharif, Bhagirath Singh, S. M. Mansour Haeryfar

**Affiliations:** 1 Department of Microbiology and Immunology, Western University, London, Ontario, Canada; 2 Department of Microbiology and Immunology, The Pennsylvania State University, Hershey, Pennsylvania, United States of America; 3 Department of Medicine, Western University, London, Ontario, Canada; 4 Department of Pathology, Western University, London, Ontario, Canada; 5 Centre for Human Immunology, Western University, London, Ontario, Canada; 6 Department of Pathobiology, University of Guelph, Guelph, Ontario, Canada; Maisonneuve-Rosemont Hospital, Canada

## Abstract

Indoleamine 2,3-dioxygenase (IDO) is a tryptophan-degrading enzyme known to suppress antitumor CD8^+^ T cells (T_CD8_). The role of IDO in regulation of antiviral T_CD8_ responses is far less clear. In addition, whether IDO controls both immunodominant and subdominant T_CD8_ is not fully understood. This is an important question because the dominance status of tumor- and virus-specific T_CD8_ may determine their significance in protective immunity and in vaccine design. We evaluated the magnitude and breadth of cross-primed T_CD8_ responses to simian virus 40 (SV40) large T antigen as well as primary and recall T_CD8_ responses to influenza A virus (IAV) in the absence or presence of IDO. IDO^−/−^ mice and wild-type mice treated with 1-methyl-D-tryptophan, a pharmacological inhibitor of IDO, exhibited augmented responses to immunodominant epitopes encoded by T antigen and IAV. IDO-mediated suppression of these responses was independent of CD4^+^CD25^+^FoxP3^+^ regulatory T cells, which remained numerically and functionally intact in IDO^−/−^ mice. Treatment with L-kynurenine failed to inhibit T_CD8_ responses, indicating that tryptophan metabolites are not responsible for the suppressive effect of IDO in our models. Immunodominant T antigen-specific T_CD8_ from IDO^−/−^ mice showed increased Ki-67 expression, suggesting that they may have acquired a more vigorous proliferative capacity *in vivo*. In conclusion, IDO suppresses immunodominant T_CD8_ responses to tumor and viral antigens. Our work also demonstrates that systemic primary and recall T_CD8_ responses to IAV are controlled by IDO. Inhibition of IDO thus represents an attractive adjuvant strategy in boosting anticancer and antiviral T_CD8_ targeting highly immunogenic antigens.

## Introduction

CD8^+^ T cells (T_CD8_) play a pivotal role in detection and elimination of virus-infected and neoplastic cells. The T_CD8_ activation cascade is triggered by professional antigen (Ag)-presenting cells (pAPCs), particularly dendritic cells (DCs), which process proteins of viral or tumor origin and load the resultant 8–11-amino acid residue-long peptides onto their major histocompatibility complex (MHC) class I molecules for presentation to T_CD8_ [1], [2]. The highly specific recognition of peptide:MHC I complexes by the T cell receptor (TCR) delivers “signal 1” to T_CD8_. Signal 1 is essential but not sufficient for optimal activation of naïve T_CD8_, which also requires a costimulatory signal (*aka.* signal 2). Signal 2 emanates from costimulatory molecules, the typical example of which is CD28 that is engaged by B7-1 (CD80) or B7-2 (CD86) present on the surface of pAPCs [3], [4].

T_CD8_ activation is achieved by direct priming and/or cross-priming. In the former pathway, T_CD8_ respond directly to virus-infected pAPCs or tumor cells that provide signal 1 and signal 2 concomitantly. However, many viruses avoid pAPCs or paralyze their Ag processing and presentation machinery. Moreover, tumor cells of non-hematopoietic origin typically do not express costimulatory molecules. T_CD8_ activation under these conditions is achieved via cross-priming, which is initiated by pAPCs that have acquired exogenous antigenic substrates from “client” cells that are not capable of priming naïve T_CD8_ on their own. We previously documented the *in vivo* significance of cross-priming in the development of antiviral and antitumor T_CD8_ responses [5]. It is noteworthy that DCs can also capture “pre-made” peptide:MHC complexes from other cells including dead or dying cells for presentation to T_CD8_. In this pathway, there is no need for further processing of the acquired complexes. The term “cross-dressing” has been coined to describe this phenomenon [2]. Although our understanding of cross-dressing in the immune system is still very limited, “cross-dressed” APCs have been implicated in inducing T_CD8_ responses to virus-infected and tumor cells as well as to cancer vaccines [6], [7].

An intriguing feature of directly primed and cross-primed T_CD8_ responses is immunodominance. This phenomenon dictates that out of thousands of peptides harbored by complex protein Ags, only a selected few elicit measurable T_CD8_ responses of varying magnitude. This establishes a dominance hierarchy among Ag-specific T_CD8_ clones. Accordingly, immunodominant peptide epitopes provoke robust T_CD8_ responses, whereas subdominant epitopes activate T_CD8_ clones occupying modest ranks in the hierarchy. Several factors have been implicated in shaping T_CD8_ hierarchies. These include the abundance of foreign gene products and the efficiency of their proteolytic degradation by APCs, the binding affinity of peptides for MHC class I molecules in the endoplasmic reticulum (ER), and the existence of epitope-specific T_CD8_ within the host T cell repertoire. We previously demonstrated that both the magnitude and the breadth of virus- and tumor-specific T_CD8_ responses are under tight control by naturally occurring regulatory T (nTreg) cells [8]. However, other immunosuppressive factors and phenomena that are likely to influence T_CD8_ immunodominance have yet to be identified.

Indoleamine 2,3-dioxygenase (IDO) is an intracellular enzyme that degrades the rare and essential amino acid tryptophan by catalyzing the oxidative cleavage of its indole ring [9]. This yields a series of biologically active catabolites collectively known as kynurenines [9], [10]. IDO may regulate immune responses by causing tryptophan starvation leading to generalized retardation of cellular proliferation [9], [11]. Alternatively or in addition, kynurenines that accumulate as a result of the enzymatic activity of IDO may exert immunosuppressive functions [12], [13].

IDO and the tryptophan catabolites it generates have been reported to inhibit T_CD8_ responses in several experimental models. For instance, overexpression of IDO in rat lung allografts was reported to reduce graft-infiltrating T_CD8_ numbers and their alloaggressive cytotoxicity [14]. IDO is expressed within various tumor microenvironments and by APCs present in tumor-draining lymph nodes [15], [16]. Importantly, IDO expression by tumors is inversely correlated with tumor-infiltrating T_CD8_ numbers in several types of malignancies including esophageal, ovarian and endometrial carcinomas, and may contribute to a poor clinical outcome [17]–[19]. Although the immunosuppressive properties of IDO in the context of cancer are relatively well understood, whether IDO controls the responsiveness of cross-primed T_CD8_ targeting multiple tumor-derived epitopes and their immunodominance hierarchies remains an open and important question.

The role of IDO in antiviral immunity is far less clear and somewhat controversial. IDO reportedly mediates the antiviral effects of interferon (IFN)-γ against dengue virus in infected DCs [20] and against hepatitis B virus in human hepatocyte-derived cells [21]. In contrast, the type I IFN response to a murine leukemia virus (MLV) was potentiated in the absence of IDO, and MLV-infected IDO-deficient mice exhibited lower viral loads and improved survival in comparison with IDO-sufficient animals [22]. Infection with influenza A virus (IAV) in mice can induce protective immunity but also paradoxically upregulates the activity of IDO in the lungs [23]. A recent study found that this activity enhances IAV-inflicted weight loss and restrains pulmonary T_CD8_ responses to IAV [24]. Whether systemic IAV-specific T cell responses are a subject of regulation by IDO is not currently understood, and any potential effect of IDO on the hierarchical pattern of antiviral T_CD8_ remains essentially unexplored. In the present study, we have addressed the above questions by examining the role of IDO in controlling *in vivo* T_CD8_ responses to simian virus 40 (SV40) large tumor Ag (T Ag) and IAV.

## Materials and Methods

### Ethics Statement

All animal procedures were approved by the Western University Animal Use Subcommittee (AUP# 2010-241) in accordance with Canadian Council on Animal Care (CCAC) guidelines.

### Mouse Strains

Wild-type (WT) C57BL/6 (B6; H-2^b^) mice and breeding pairs of IDO gene knockout (IDO^−/−^) mice on B6 background were purchased from Charles River Canada Inc. (St. Constant, QC) and Jackson Laboratories (Bar Harbor, ME), respectively. Adult mice closely matched for age and sex were used in all experiments.

### Cell Lines

The SV40-transformed mouse cell lines C57SV (H-2^b^) [25] and KD2SV (H-2^d^) [26] were provided by Drs. Jack Bennink and Jonathan Yewdell (National Institutes of Health) and maintained in DMEM supplemented with 5% fetal bovine serum (FBS). The immature mouse dendritic cell line DC2.4 (H-2^b^) [27] was obtained from Dr. Kenneth Rock (University of Massachusetts). DC2.4 cells and the mouse thymoma cell line EL4 (ATCC TIB-39) were grown in RPMI 1640 medium containing 10% FBS, nonessential amino acids, glutamax and 1 mM sodium pyruvate, which will hereafter be referred to as complete medium.

### Peptides and Chemicals

Peptides used in this investigation (Table 1) were >95% pure. They were generously provided by Drs. Jack Bennink and Jonathan Yewdell (National Institutes of Health). Stock solutions of all peptides were prepared at 1 mM in DMSO and stored at −30°C. Each peptide was used at a final concentration of 500 nM in our assays. 1-Methyl-D-tryptophan (1-D-MT) was purchased from Sigma (Mississauga, ON). The powder was dissolved at 5 mg/mL in 0.1 M NaOH followed by drop-wise addition of 12 M HCl to generate a neutral solution, which was then filter-sterilized and injected as indicated. L-kynurenine (L-Kyn), also from Sigma, was dissolved at 10 mg/mL in PBS shortly before injection.

**Table 1:** Peptides used in this study.

Ag Source	Epitope	Designation	Sequence	MHC Restriction
SV40^a^ Large T Ag	T Ag_206–215_	Site I	SAINNYAQKL	H-2D^b^
SV40 Large T Ag	T Ag_223–231_	Site II/III	CKGVNKEYL	H-2D^b^
SV40 Large T Ag	T Ag_404–411_	Site IV	VVYDFLKC	H-2K^b^
SV40 Large T Ag	T Ag_489–497_	Site V	QGINNLDNL	H-2D^b^
IAV^b^ Nucleoprotein	NP_366–374_	NP_366_	ASNENMETM	H-2D^b^
IAV Acid Polymerase	PA_224–233_	PA_224_	SSLENFRAYV	H-2D^b^
IAV PB1-F2 Protein	PB1-F2_62–70_	PB1-F2_62_	LSLRNPILV	H-2D^b^
IAV PB1 Polymerase	PB1_703–711_	PB1_703_	SSYRRPVGI	H-2K^b^
IAV PB2 Polymerase	PB2_198–206_	PB2_198_	ISPLMVAYM	H-2K^b^
IAV Nonstructural Protein 2	NS2_114–121_	NS2_114_	RTFSFQLI	H-2K^b^
IAV Matrix Protein 1	M1_128–135_	M1_128_	MGLIYNRM	H-2K^b^
HSV-1^c^ Glycoprotein B	gB_498–505_	gB_498_	SSIEFARL	H-2K^b^

a SV40: simian virus 40;

b IAV: influenza A virus (Puerto Rico/8/34 strain);

c HSV-1: herpes simplex virus-1.

### Immunization and Treatment Protocols

Once confluent, SV40-transformed tumor cells were trypsinized, thoroughly washed, filtered, resuspended in sterile PBS and injected at 2×10^7^ cells per mouse intraperitoneally (*i.p.*).

The PR8 (Puerto Rico/8/34, H1N1) and X31 (H3N2) strains of influenza A virus (IAV) were propagated in 10-day-old embryonated chicken eggs. For *i.p.* inoculation of PR8, infectious allantoic fluid was diluted 1∶2 in PBS and injected at 500 µL per mouse, which approximates ∼600 hemagglutinating units. In our respiratory flu infection model, 50 µL sterile PBS containing 6–8 plaque-forming units of PR8 was administered intranasally (*i.n.*) to each anesthetized mouse. Animals were carefully monitored afterwards to avoid significant weight loss and morbidity. To examine recall anti-IAV T_CD8_ responses, we used an established prime-boost immunization protocol in which mice were inoculated *i.p.* with PR8 (H1N1), and challenged one month later with the X31 reassortant virus (H3N2) that was also injected *i.p.* [28].

In several experiments, mice received four *i.p.* doses of 1-D-MT, 2.5 mg per dose per animal, or equal volumes of ion-matched pH-adjusted PBS solution as vehicle, on days −2, −1, +1 and +2. C57SV cells IAV were injected into these animals on day 0. In separate experiments, C57SV- or IAV-inoculated mice were treated with three *i.p.* doses of L-Kyn, 10 mg each, or PBS, on days 0, +1 and +2.

To inactivate naturally occurring regulatory T (nTreg) cells, a single 1-mg dose of the PC61 anti-CD25 monoclonal antibody (mAb) (Bio X Cell, West Lebanon, NH) was administered *i.p.* 3 days before immunization with C57SV cells [8].

### Cytofluorimetric Analyses

Unless otherwise stated, all fluorochrome-labeled Abs and isotype controls used in this study were from eBioscience (San Diego, CA) or BD Pharmingen (San Jose, CA). The frequency of splenic nTreg cells was determined based on their co-expression of CD4, CD25 and forkhead box P3 (FoxP3) using a kit from e-Bioscience.

T_CD8_ responses were mainly assessed by the sensitive and highly quantitative method of intracellular cytokine staining (ICS) for interferon (IFN)-γ as we previously described [8]. In brief, 9 days after immunization with SV40-transformed tumor cells, 7 days after *i.p.* inoculation of IAV, or 9 days after *i.n.* infection with IAV, time points at or around which corresponding T_CD8_ responses reach their maximum [8], [29], [30], mice were euthanized for their spleen. Erythrocyte-depleted splenocytes were resuspended in complete medium and stimulated *ex vivo*, as appropriate, with C57SV cells, KD2SV cells, PR8-infected DC2.4 cells [8], or synthetic peptides corresponding to T Ag- or IAV-derived epitopes, which are listed in Table 1. The H-2^b^-restricted immunodominant peptide derived from herpes simplex virus (HSV)-1, namely gB_498_, served as a control. After 2-hour incubation at 37°C, 10 µg/mL of brefeldin A (Sigma) was added to retain IFN-γ in the ER of activated T_CD8_. Cultures were continued for an additional 3–4 hours before cells were spun, washed and incubated on ice with 5 µg/mL Fc Block (anti-CD16/CD32 mAb) to prevent nonspecific, FcR-mediated binding of Abs. Cells were then stained for surface CD8, washed, fixed with 1% paraformaldehyde, washed again and permeabilized with 0.1% saponin to enable staining for intracellular IFN-γ. A BD FACSCanto II flow cytometer and FlowJo software (Tree Star, Ashland, OR) were used for data acquisition and analysis, respectively. The percentage and/or absolute number (per spleen) of IFN-γ^+^ cells were determined after live gating on CD8^+^ events. Our gating strategy for detection of immunodominant T Ag-specific T_CD8_ by ICS is illustrated in Fig. S1.

In several experiments, an Alexa Fluor 488-conjugated anti-human Ki-67 mAb (clone B56, BD Pharmingen) and an anti-mouse C/EBP-homologous protein (CHOP) mAb (clone L63F7) were included in our ICS protocol for intracellular staining of Ki-67 and CHOP in Ag-specific T_CD8_. The B56 mAb reacts with mouse Ki-67. Anti-CHOP mAb was purchased from Cell Signaling Technology (Beverly, MA) and conjugated with Alexa Fluor 488 using a mAb labeling kit from Invitrogen (Burlington, ON).

In a limited number of experiments, T Ag-specific T_CD8_ were identified using MHC class I tetramers. H-2K^b^/IV and H-2D^b^/I tetramer reagents were prepared and used as we previously described [31].

### Enzyme-linked Immunospot (ELISPOT) Assays

Granzyme B (GrB)-secreting cells were spotted and enumerated using ELISPOT kits and assay protocols obtained from R&D Systems (Minneapolis, MN). Briefly, 96-well PVDF membrane plates were coated overnight at 4°C with an anti-GrB capture mAb. Membranes were subsequently blocked for 2 hours and washed before erythrocyte-depleted splenocytes from T Ag-primed WT and IDO^−/−^ mice were seeded at 3×10^5^ cells/well and stimulated with indicated peptides. Concanavalin A (ConA) was used as a positive control at a final concentration of 5 µg/mL. The plates were incubated overnight at 37°C and 6% CO_2_ in a humidified atmosphere. Cells were then removed and the plates were washed before a biotin-labeled polyclonal Ab detecting mouse GrB was added to the wells. After overnight incubation at 4°C, the plates were washed and 100 µL of streptavidin-alkaline phosphatase (1∶60 in PBS/1% BSA) was added to each well. The plates were incubated at room temperature for an additional 2 hours and spots were visualized with BCIP/NBT (5-bromo-4-chloro-3′ indolylphosphate p-toluidine salt and nitro blue tetrazolium chloride in organic solvent) and subjected to automated evaluation using an ImmunoSpot S5 UV Analyzer (Cellular Technology Ltd., Cleveland, OH).

### Cytotoxicity Assay

Cytotoxic T lymphocyte (CTL) induction in WT and IDO^−/−^ mice was assessed on day 9 post-priming by standard ^51^Chromium (^51^Cr) release assays. Splenocytes were prepared and used at indicated effector:target ratios against ^51^Cr-labeled EL4 target cells seeded at 10^4^ cells/well of a U-bottom microplate. EL4 cells were pre-sensitized with 100 nM T Ag-derived peptides. After 8-hour incubation at 37°C, the plates were spun for 5 minutes at 400×*g* and a 100-µL aliquot of supernatant was harvested from each well. The ^51^Cr activity of the samples was determined by a γ counter, and the following formula was used to calculate specific lysis of the target cells: % specific lysis = [(ER−SR)/(TR−SR)]×100, where ER (experimental release) is obtained from wells containing both effector and target cells, whereas SR (spontaneous release) and TR (total release) correspond to wells receiving target cells plus medium or target cells plus 1% Triton X-100, respectively.

### T Cell Proliferation

Splenocytes from WT and IDO^−/−^ mice were seeded at 4×10^5^ cells/well of a U-bottom microplate. Cells were left untreated or stimulated with a 1∶20 dilution of hybridoma supernatant containing anti-CD3ε (clone 145-2C11) approximating 0.25 µg/mL of this mAb, 5 µg/mL of phytohemagglutinin (PHA) or 1 µg/mL of ConA. Plates were kept at 37°C and 6% CO_2_ for 72 hours. Cells were pulsed with 1 µCi of tritiated thymidine ([^3^H]TdR) for the final 18 hours of the cultures. Cultures were then harvested onto glass fiber filter mats and [^3^H]TdR uptake was determined by liquid scintillation counting as a measure of T cell proliferation.

### DC Preparation and *in vitro* nTreg Suppression Assays

Bone marrow-derived DCs (BMDCs) were generated by culturing marrow cells flushed out of femurs and tibias with recombinant granulocyte-macrophage colony-stimulating factor (GM-CSF) and interleukin (IL)-4 (10 ng/mL each). DCs were magnetically purified using an EasySep mouse CD11c positive selection kit from StemCell Technologies (Vancouver, BC).

nTreg cells from WT and IDO^−/−^ mice were magnetically purified using a mouse CD4^+^CD25^+^ regulatory T cell isolation kit from Miltenyi Biotec (Auburn, CA). Varying numbers of nTreg cells were then co-cultured with 1×10^5^ conventional (CD4^+^CD25^−^) T cells, 2×10^4^ γ-irradiated (3000 RADs) BMDCs as APCs, and a 1∶20 dilution of hybridoma supernatant containing anti-CD3ε (clone 145-2C11). T cell proliferation was measured by [^3^H]TdR incorporation as described above.

### Statistical Analysis

Statistical comparisons were performed with the aid of GraphPad Prism software. *, ** and *** denote *p*<0.05, *p*<0.01 and *p*<0.001, respectively.

## Results

### The Cross-primed T_CD8_ Response to the T Ag’s Most Immunodominant Epitope is Augmented in IDO^−/−^ Mice

The immunoregulatory function of IDO mediates tolerance and contributes to immune suppression in a variety of settings including in animal models of cancer [9], [32]. IDO is known to inhibit T cell proliferation, differentiation and effector functions [15], [33], [34]. However, whether cross-primed T_CD8_ responses to cell-associated tumor Ags are subject to IDO regulation is not clear. Moreover, whether immunodominant and subdominant tumor Ag-specific T_CD8_ are equally controlled by IDO is unknown. To address these questions, we investigated *in vivo* T_CD8_ responses of WT and IDO^−/−^ mice to SV40 large T Ag, a clinically relevant oncoprotein that mediates neoplastic transformation of various mammalian cell types [35].

**Figure 1:**
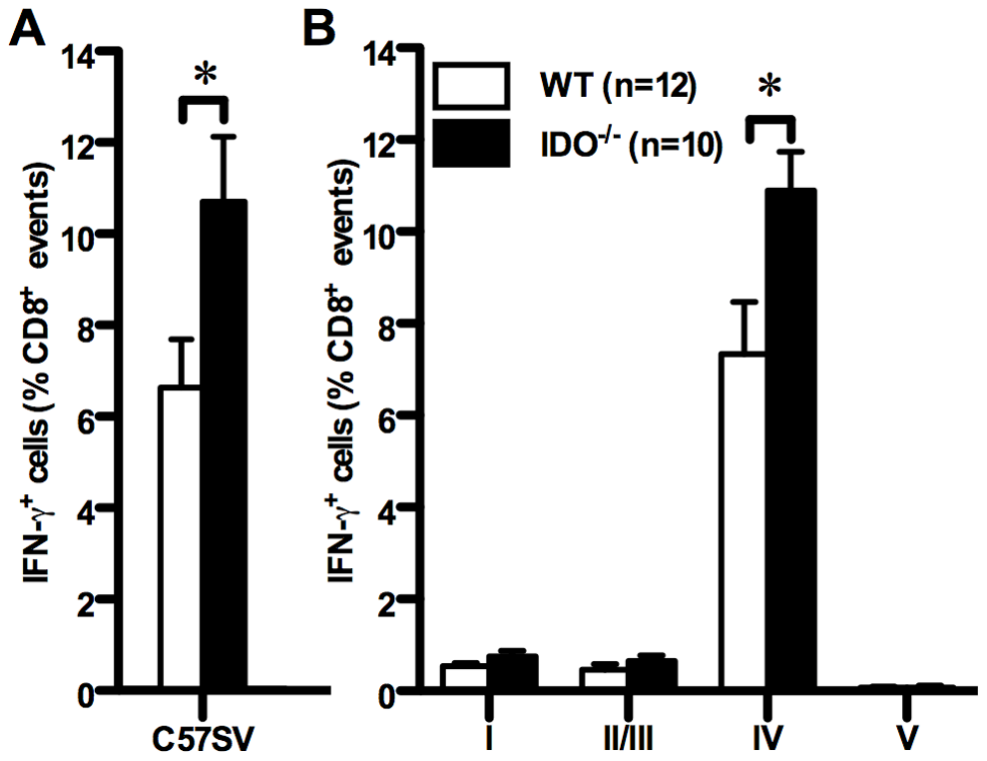
Genetic deficiency of IDO enhances the T_CD8_ response to T Ag’s most immunodominant epitope. WT and IDO^−/−^ mice were injected *i.p.* with syngeneic, SV40-transformed C57SV cells. Nine days later, splenic T_CD8_ were examined *ex vivo* for IFN-γ accumulation following brief restimulation (of 5 hours in duration) with C57SV cells used at 2×10^5^ cells/well (A) or synthetic peptides corresponding to T Ag-derived epitopes (B). Background obtained from wells receiving no peptide was subtracted and values are expressed as mean ± standard error of the mean (SEM) of multiple mice per group (n = 12 and n = 10 for WT and IDO^−/−^ mice, respectively) pooled from independent experiments yielding similar results.

In B6 mice, T Ag-specific T_CD8_ recognize and target four H-2^b^-restricted peptide epitopes, termed sites I, II/III, IV, and V (Table 1), which fall into the following immunodominance hierarchy: site IV >> I ≥ II/III >> V [5], [8], [31]. To examine the contribution of IDO to this hierarchy, we inoculated WT and IDO^−/−^ B6 mice with C57SV cells, a syngeneic SV40-transformed tumor cell line that expresses T Ag. Nine days later, T_CD8_ specific for sites I, II/III and IV were readily detectable in the spleens by ICS for IFN-γ and followed the characteristic pattern of T Ag-specific immunodominance (Fig. 1). No reactivity was detectable against gB_498_, an irrelevant peptide that was used as a control (data not shown). IDO deficiency augmented the bulk T Ag-specific response as judged by the increased frequency of splenic T_CD8_ producing IFN-γ following *ex vivo* exposure to C57SV cells (Fig. 1A). Although ablation of IDO led to similar fold increases in average frequencies of IFN-γ^+^ T_CD8_ recognizing certain T Ag-derived epitopes, the only difference that reached statistical significance was linked to the T Ag’s most immunodominant epitope (*i.e.*, site IV) (Fig. 1B and Table S1). It is noteworthy that a site V-specific response becomes detectable only in the absence of T_CD8_ priming by other T Ag epitopes [31], [36], [37], and lack of IDO failed to elevate this immunorecessive response to an appreciable level (Fig. 1B). We also found moderate increases in absolute numbers of T Ag-specific, IFN-γ-producing T_CD8_ in the spleen of IDO^−/−^ mice although statistical significance was not reached for these comparisons (Fig. S2). Of note, the mean fluorescence intensity (MFI) of IFN-γ staining was very similar between WT and IDO^−/−^ T_CD8_ recognizing site IV and other T Ag epitopes (Fig. S3).

In a limited number of experiments, we quantitated T Ag-specific T_CD8_ independently of their ability to synthesize IFN-γ. We inoculated WT and IDO^−/−^ mice with C57SV cells and determined the frequencies of their site I- and site IV-specific T_CD8_ using H-2D^b^/I and H-2K^b^/IV tetramer reagents, respectively. For both epitopes, splenic tetramer-reactive T_CD8_ levels were comparable between WT and IDO^−/−^ mice (Fig. S4). Therefore, when taken together, data obtained from ICS assays and tetramer staining indicate that IDO suppresses “functional” T Ag-specific T_CD8_ responses.

**Figure 2:**
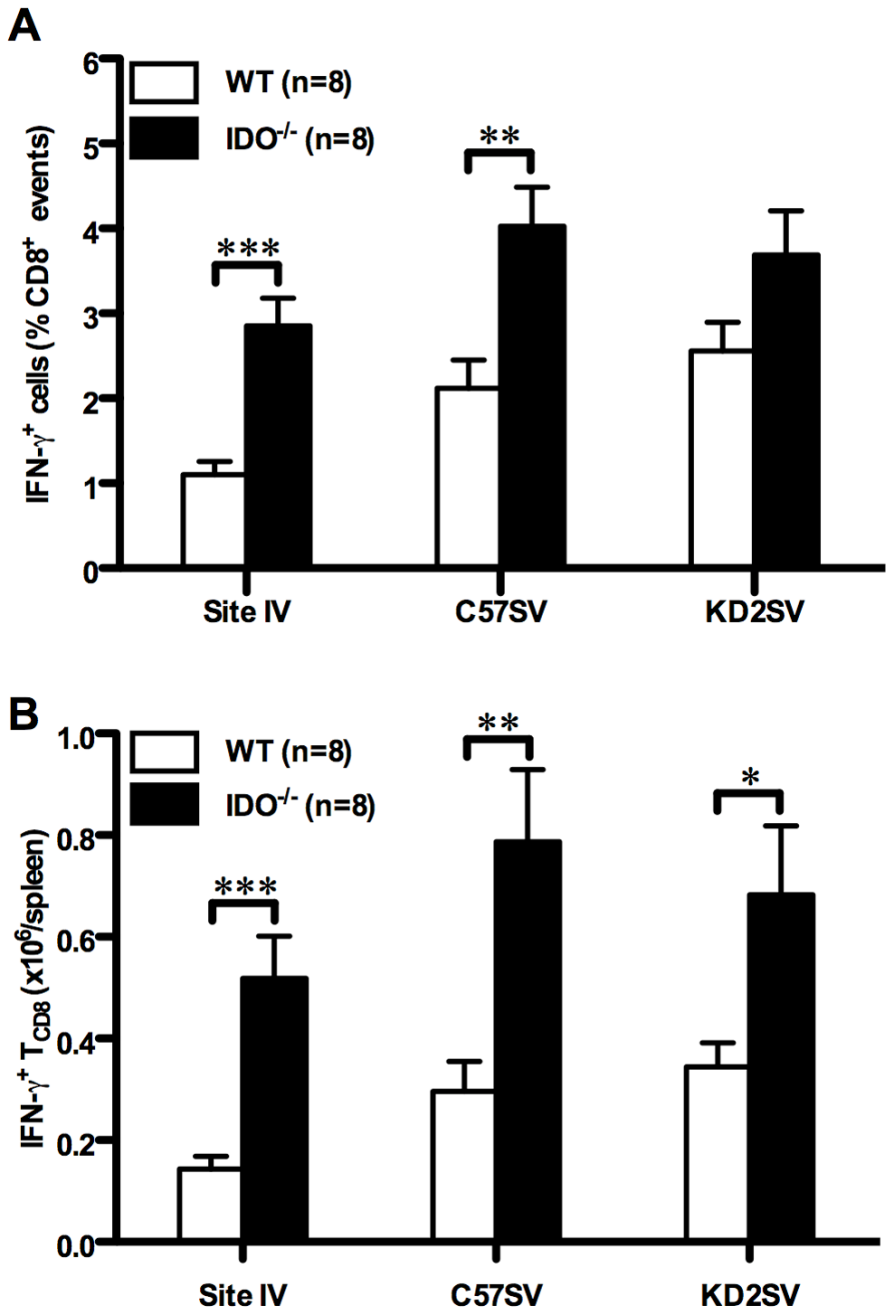
The cross-primed T_CD8_ response to T Ag is augmented in the absence of IDO. WT and IDO^−/−^ mice were injected *i.p.* with allogeneic T Ag^+^ KD2SV cells. Nine days later, the frequencies (A) and absolute numbers (B) of T Ag-specific T_CD8_ recognizing site IV, total T Ag-specific T_CD8_ that synthesize IFN-γ after incubation with C57SV cells, and total alloreactive T_CD8_ that produce IFN-γ after incubation with KD2SV cells were determined by ICS as described in Materials and Methods. Background obtained from wells receiving no peptide was subtracted and values are presented as mean ± SEM of 8 mice/group pooled from independent experiments.

T Ag-specific responses in our model are induced through the cross-priming pathway. This is because C57SV cells are of fibroblastic origin, not pAPCs, and do not express classical costimulatory molecules such as CD80 and CD86 [8]. Therefore, they are presumably unable to directly activate naïve T_CD8_. Furthermore, they are transformed with subgenomic fragments of SV40 and fail to generate SV40 virions [5], [8]. This eliminates the possibility that the ensuing T_CD8_ responses are due to the infection of host pAPCs. Nevertheless, to more definitively explore the role of IDO in negative regulation of cross-primed T_CD8_ responses, we injected WT and IDO^−/−^ B6 (H-2^b^) mice with the T Ag^+^, MHC-mismatched (H-2^d^) kidney epithelial tumor cell line KD2SV. Consistent with our previous reports, in addition to simultaneous allostimulation, strong T Ag-specific T_CD8_ responses are generated in KD2SV-injected B6 mice, which rely exclusively on cross-priming according to the rule of MHC restriction [5], [8], [38]. We found a dramatic increase in both the percentage and the absolute number of splenic site IV-specific T_CD8_ in IDO^−/−^ mice that were injected with KD2SV cells (Fig. 2A and Fig. 2B). The bulk T Ag-specific and alloreactive T_CD8_ responses, manifested by *ex vivo* reactivity with C57SV and KD2SV cells respectively, were also enhanced in the absence of IDO (Fig. 2). By contrast, the site V-specific response was comparable in WT and IDO mice. In addition, although the observed increases in the absolute number of site I- and site II/III-specific T_CD8_ in IDO^−/−^ mice were statistically significant, these changes were not as marked as that found for site IV (compare *p* values in Table S2). The above data collectively demonstrate that IDO inhibits functional antitumor T_CD8_ responses induced *in vivo* through cross-priming and that the suppressive effect of IDO is pronounced against immunodominant tumor-derived epitopes.

### Pharmacological Inhibition of IDO Leads to an Enhanced T_CD8_ Response to Site IV

To ascertain that the increased site IV-specific T_CD8_ response in IDO^−/−^ mice results from the absence of IDO activity, as opposed to possible intrinsic changes (*e.g.*, T_CD8_ repertoire modifications) caused by the genetic deficiency of IDO, we tested the effect of 1-methyl-tryptophan (1-MT) in our model. 1-MT is a potent pharmacological inhibitor of IDO [39] that has been employed in both preclinical and clinical studies to boost antitumor immunity [15], [40], [41]. 1-MT exists as two stereoisomers, 1-D-MT and 1-L-MT, which exhibit cell type-specific variations in their activity [42]. We used 1-D-MT because this stereoisomer is reportedly more effective in relieving T cell suppression mediated by IDO-expressing DCs *in vitro* and as an anticancer agent in chemoimmunotherapy of transplantable melanoma and transplantable and autochthonous breast cancer in mice [42].

**Figure 3:**
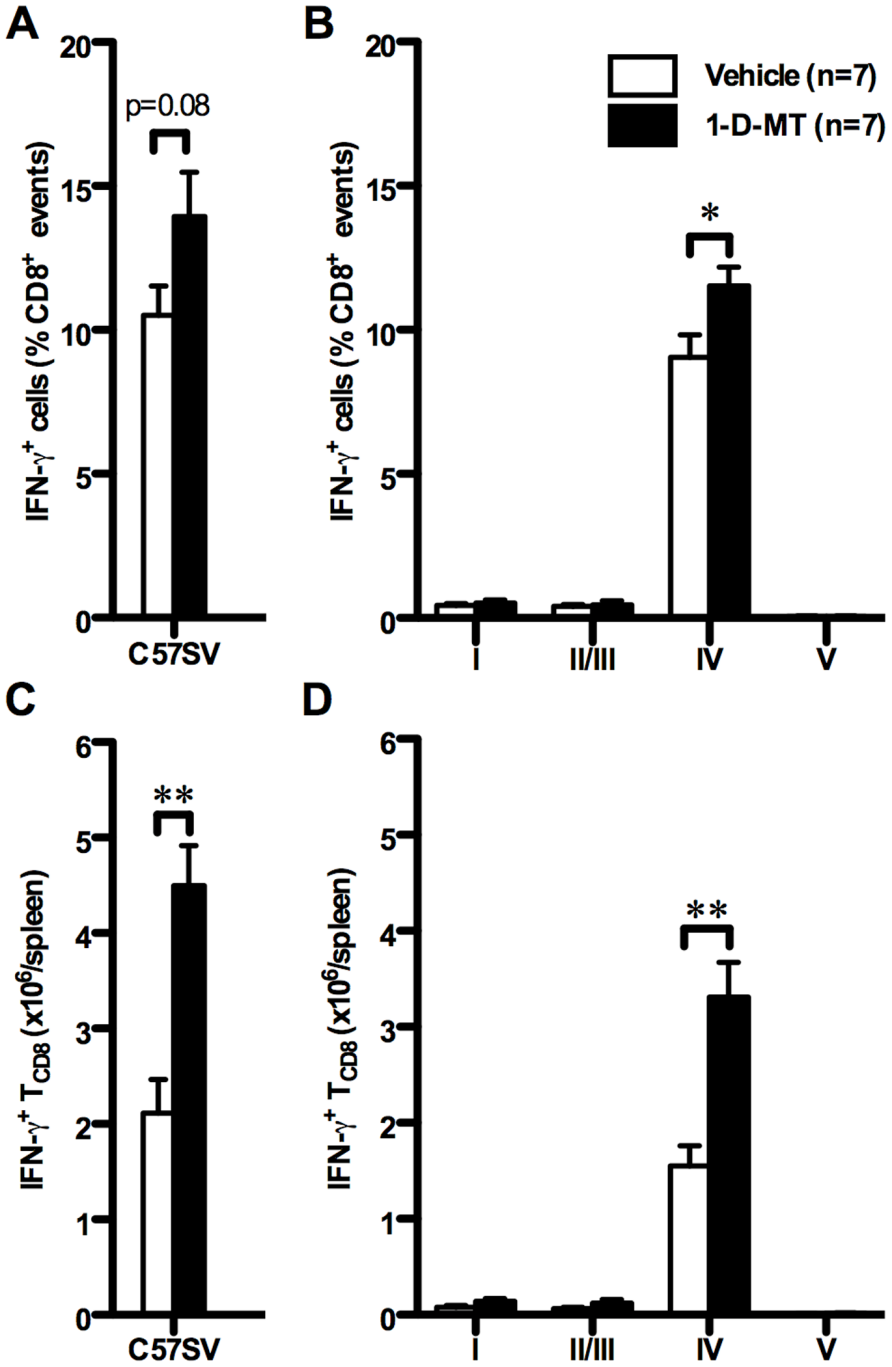
Pharmacological inhibition of IDO amplifies the T_CD8_ response to T Ag’s most immunodominant epitope. WT mice were treated with 1-D-MT (10 mg/mouse total) or vehicle and injected *i.p.* with C57SV cells. Nine days later, splenic T_CD8_ were examined *ex vivo* for IFN-γ accumulation following restimulation with C57SV cells (A and C) or synthetic peptides corresponding to T Ag epitopes (B and D). T Ag-specific T_CD8_ frequencies were determined after subtracting background and expressed as mean ± SEM of 7 mice per group (A and B). These values were used to calculate the absolute number of T Ag-specific T_CD8_ present within each spleen (C and D).

Treatment of WT mice with 1-D-MT led to a strong bulk T Ag-specific response (Fig. 3A and Fig. 3C) and significantly increased the frequency and absolute number of splenic site IV-specific IFN-γ^+^ T_CD8_ (Fig. 3B, Fig. 3D and Table S3). In contrast, T_CD8_ responses to subdominant T Ag epitopes were not significantly affected by 1-D-MT treatment (Fig. 3B, Fig. 3D and Table S3), which again recapitulates the response pattern observed in IDO^−/−^ mice. In subsequent experiments, administration of 1-D-MT to IDO^−/−^ mice failed to further increase the site IV-specific response beyond the level detected in vehicle-treated IDO^−/−^ mice or 1-D-MT-treated WT mice (data not shown). Therefore, the deficiency and pharmacological inhibition of IDO appear to work through the same mechanism(s) to enhance the vigor of the immunodominant T_CD8_ response in the T Ag system.

### T Ag-specific T_CD8_-mediated Cytotoxicity is Augmented in the Absence of IDO

**Figure 4:**
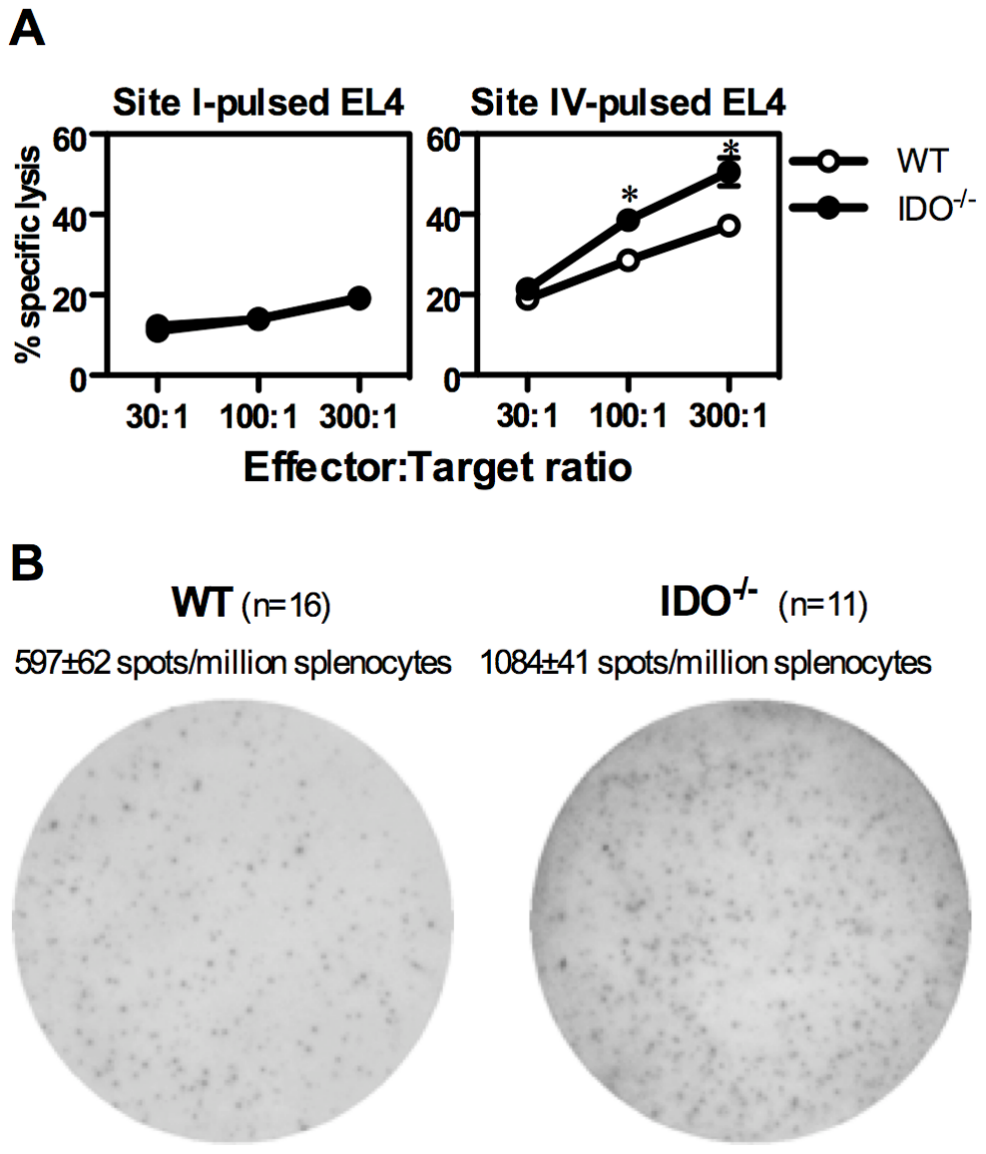
T Ag-specific T_CD8_-mediated cytotoxicity and granzyme B secretion in the presence and absence of IDO. (A) WT and IDO^−/−^ mice were injected *i.p.* with C57SV cells. Nine days later, splenocytes from each group, typically consisting of 2–3 mice each, were pooled and used at indicated effector:target ratios against ^51^Cr-labeled EL4 target cells sensitized with indicated T Ag-derived peptides. Each data point represents mean ± standard deviation of quadruplicate samples. Similar results were obtained in 2 independent experiments. (B) Splenocytes from T Ag-primed WT and IDO^−/−^ mice were restimulated *ex vivo* with site IV, and granzyme B (GrB)-secreting cells were enumerated by ELISPOT as described in Materials and Methods. A representative ELISPOT well for each group is illustrated along with mean ± SEM of GrB spots per 10^6^ splenocytes for 16 WT and 11 IDO^−/−^ mice.

IFN-γ production is but one of several important T_CD8_ properties that can differ considerably among distinct functional T_CD8_ subsets. To extend our findings to CTL function, we examined the effect of IDO on T_CD8_-mediated T Ag-specific cytotoxicity. Nine days after inoculation of WT and IDO^−/−^ mice with C57SV cells, we determined the capacity of their splenocytes *ex vivo* to kill ^51^Cr-labeled, natural killer cell-resistant EL4 thymoma cells [43] pulsed with synthetic peptides corresponding to T Ag epitopes. The tumoricidal activity of IDO^−/−^ splenocytes against site IV-pulsed EL4 cells was higher than that exhibited by WT splenocytes (Fig. 4A). By contrast, lack of IDO failed to augment specific lysis of site I- and site II/III-displaying cells or to induce detectable cytotoxicity against site V-sensitized cells (Fig. 4A and data not shown). Therefore, although the absolute order of the T Ag-specific T_CD8_ hierarchy was maintained in IDO^−/−^ mice, site IV-specific killing was enhanced. This finding was reproducible in our *in vivo* cytotoxicity experiments that assayed for the splenic CTL function of C57SV-primed mice against naïve splenocytes pulsed with site IV, which we previously described [8].

The enhanced cytotoxic effector function exhibited by IDO^−/−^ splenocytes was consistent with a numerical increase in site IV-reactive GrB-secreting cells following inoculation of IDO^−/−^ mice with C57SV cells (Fig. 4B). In contrast, comparable numbers of GrB-secreting cells could be spotted among WT and IDO^−/−^ splenocytes after *in vitro* stimulation with the T cell mitogen ConA [940±77 and 1,050±97 spot-forming cells per million splenocytes for WT mice and IDO^−/−^ mice, respectively (n = 11 each)]. Therefore, lack of IDO increases the number of T-Ag-specific, GrB-secreting T_CD8_ without altering the non-specific T cell response to a mitogenic lectin.

### The Frequency and Suppressor Function of nTreg Cells are Intact in IDO^−/−^ Mice

**Figure 5:**
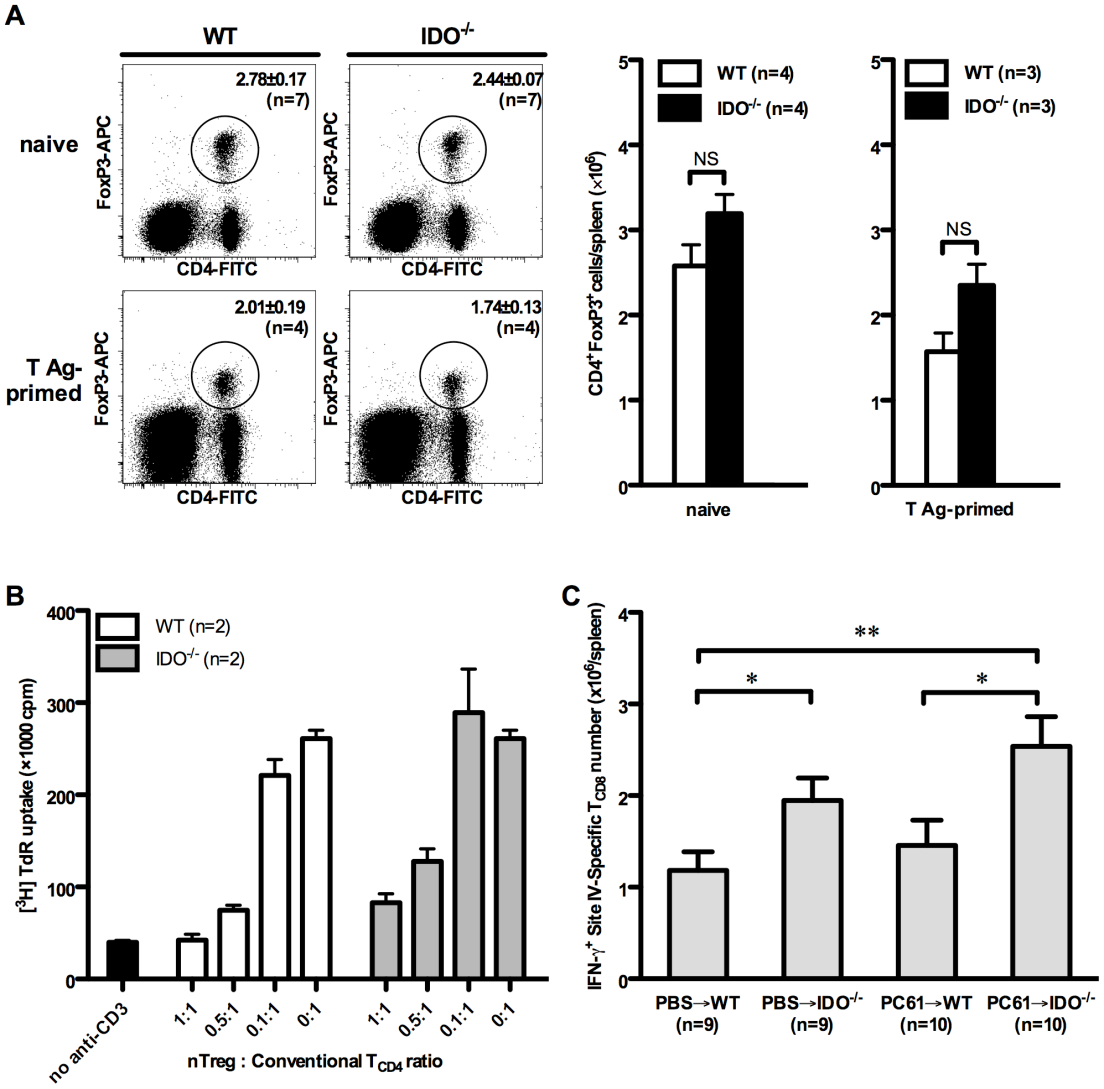
nTreg cells do not mediate the suppressive effect of IDO on the site IV-specific response. (A) Splenocytes from indicated numbers of naïve WT and IDO^−/−^ mice were stained for surface CD4 and intracellular FoxP3. In separate experiments, WT and IDO^−/−^ mice were inoculated with C57SV cells followed, 9 days later, by cytofluorimetric determination of their splenic nTreg cell frequencies, which were used to calculate the absolute number of nTreg cells within each spleen. Representative FACS plots are shown in addition to mean nTreg cell frequencies and absolute numbers ± SEM for each group. (B) WT CD4^+^CD25^−^ conventional T cells were co-cultured with γ-irradiated bone marrow-derived DCs and stimulated with an anti-CD3 mAb in the presence of varying numbers of CD4^+^CD25^+^ nTreg cells magnetically purified from WT and IDO^−/−^ mice. T cell proliferation was measured by tritiated thymidine incorporation after 72 hours. (C) WT and IDO^−/−^ mice were injected with an anti-CD25 mAb (clone PC61) or PBS 3 days before they were inoculated with C57SV cells. Nine days later, site IV-specific T_CD8_ were enumerated by ICS for IFN-γ. Values are presented as mean ± SEM for indicated numbers of mice per group pooled from 3 independent experiments.

IDO can promote naïve T cell differentiation into FoxP3^+^ Treg cells *in vitro* [34], indicating a link between these two potent mechanisms of immunosuppression. We previously demonstrated that nTreg cells shape the T_CD8_ hierarchy in the very model employed in the current work [8]. In fact, Ab-mediated depletion/inactivation of nTreg cells resulted in an augmented response to site IV [8] in a manner strikingly similar to what we have observed in IDO^−/−^ or 1-D-MT-treated WT mice (Fig. 1 and Fig. 3). Therefore, we asked whether the increased response to site IV in IDO^−/−^ mice results from numerical or functional insufficiencies in their nTreg compartment. We found that naïve WT and IDO^−/−^ mice had similar percentages and absolute numbers of CD4^+^FoxP3^+^ (Fig. 5A) or CD4^+^CD25^+^ cells in their spleen (Fig. S5). This was true also for T Ag-primed WT and IDO^−/−^ mice at the peak of their response on day 9 (Fig. 5A and Fig. S5).

In the next series of experiments, we used a standard suppression assay to compare the suppressor function of nTreg cells obtained from naïve WT and IDO^−/−^ mice. These experiments demonstrated that nTreg cells isolated from IDO^−/−^ mice are able to inhibit anti-CD3-driven conventional T cell proliferation although they appear to be slightly weaker than their WT counterparts in this capacity (Fig. 5B).

To demonstrate that the increased site IV-specific T_CD8_ response in IDO^−/−^ mice is not caused by a possible nTreg cell functional defect in these animals, we injected WT and IDO^−/−^ mice with an anti-CD25 mAb (clone PC61) that is known to deplete or inactivate nTreg cells [8] before they were injected with C57SV cells. Control groups received PBS or a rat IgG1 isotype control (Fig. 5C). We confirmed that unlike PBS or irrelevant rat IgG1, treatment with PC61 substantially reduces both the frequency and the absolute number of CD4^+^FoxP3^+^ nTreg cells in the spleen (Fig. S6). Furthermore, this reduction was comparable in WT and IDO^−/−^ mice (Fig. S6). As expected, IDO^−/−^ mice exhibited a stronger response to site IV compared to WT mice, which was further enhanced by PC61 treatment (Fig. 5C). Therefore, lack of IDO boosts the site IV-specific response by an nTreg cell-independent mechanism.

### Treatment with L-Kyn Increases Rather than Decreases the Site IV-specific T_CD8_ Response

**Figure 6:**
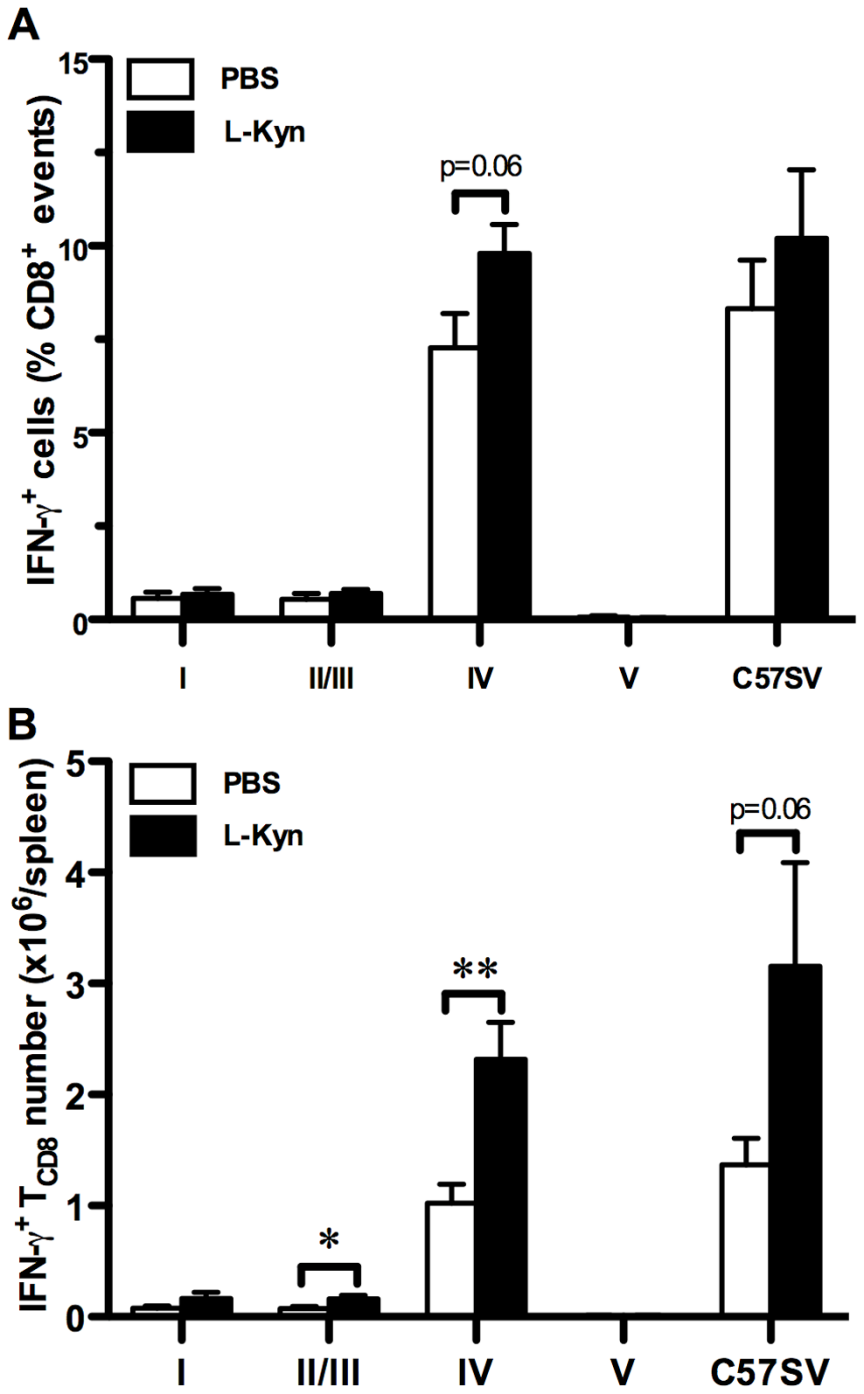
Treatment with L-Kynurenine fails to inhibit *in vivo* T_CD8_ responses to T Ag. WT mice were treated with L-Kyn (30 mg total as described in Materials and Methods) or PBS, and injected *i.p.* with C57SV cells. Nine days later, splenic T_CD8_ were examined for IFN-γ accumulation following restimulation with C57SV cells or synthetic peptides corresponding to T Ag epitopes. T Ag-specific T_CD8_ frequencies were determined after subtracting background and expressed as mean ± SEM of 6 L-Kyn-treated and 8 vehicle-treated mice (A). These values were used to calculate the absolute number of T Ag-specific T_CD8_ present within each spleen (B).

Although tryptophan starvation has immunological consequences, there is enough evidence to suggest that tryptophan metabolites generated by IDO exert immunomodulatory properties of their own and could be directly responsible for some of the effects ascribed to IDO [12], [13], [34], [44], [45]. Therefore, we asked whether the missing accumulation of such metabolites in IDO^−/−^ mice may be linked to their robust response to site IV. To mimic this scenario, we injected WT mice with 3 daily doses of L-kynurenine (L-Kyn), the most immediate stable downstream metabolite of L-tryptophan, starting on the day of C57SV cell inoculation. Contrary to our expectations, L-Kyn administration at 10 mg per day not only failed to decrease the site IV-specific response, but instead increased the frequency and absolute number of site IV-specific T_CD8_ (Fig. 6A, Fig. 6B and Table S4), thus simulating the genetic deficiency or pharmacological inhibition of IDO. Therefore, the observed suppression of site IV-specific T_CD8_ by IDO appears not to involve L-Kyn.

### Site IV-specific T_CD8_ in IDO^−/−^ Mice Express High Levels of Ki-67

**Figure 7:**
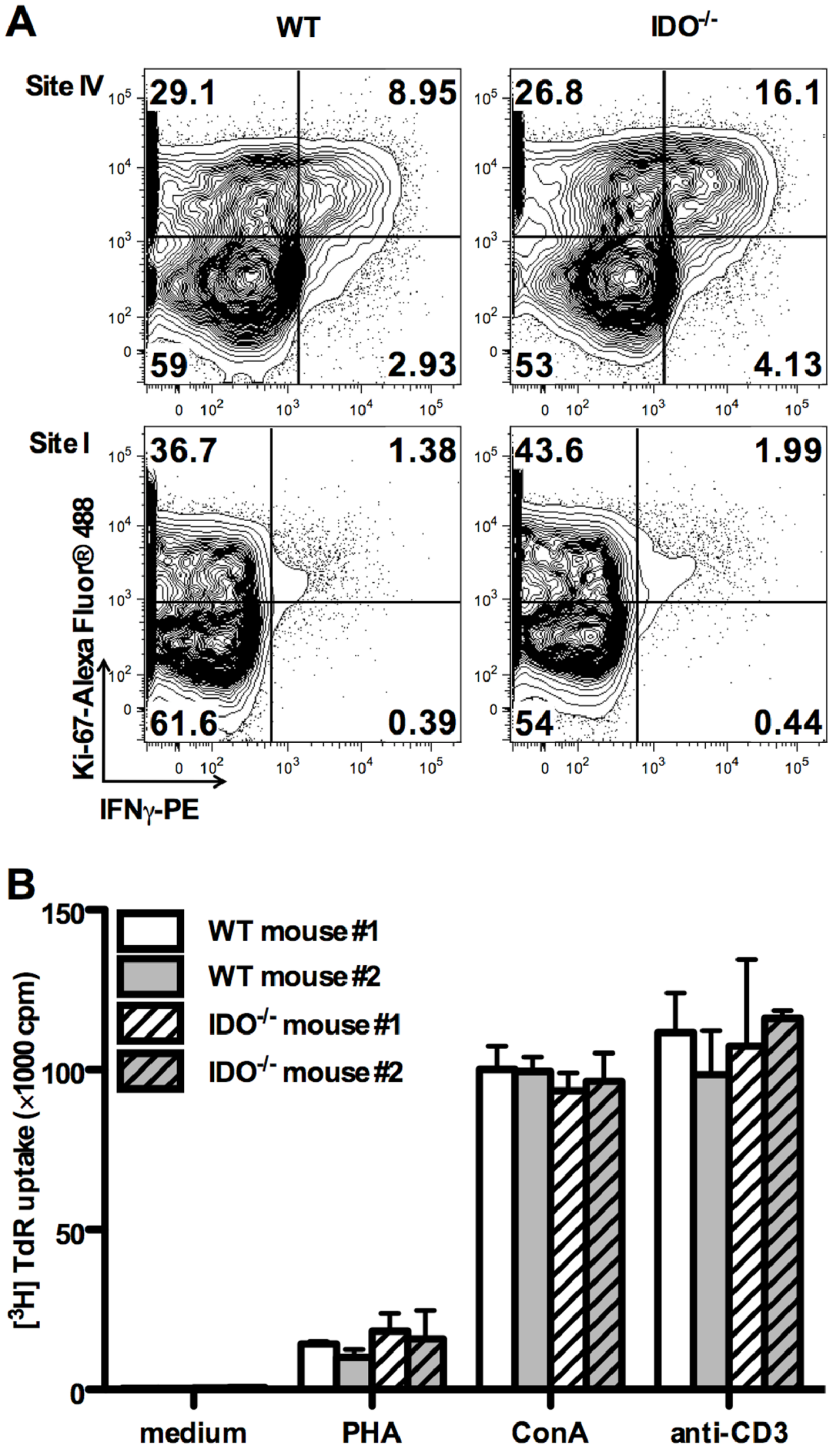
Ki-67 expression by T Ag-specific T_CD8_ and non-specific proliferative responses of IDO^−/−^ T cells. (A) Splenocytes from WT and IDO^−/−^ mice inoculated with C57SV cells were restimulated for 5 hours with synthetic peptides corresponding to site I or site IV. Cells were then stained for surface CD8, intracellular IFN-γ and intracellular Ki-67 as described in Materials and Methods. Representative FACS plots after live gating on CD8^+^ events are shown. Quadrants’ positions were set based on staining with an isotype control. (B) WT and IDO^−/−^ splenocytes were left untreated or stimulated with PHA, ConA or a mitogenic anti-CD3 mAb. T cell proliferation was measured by tritiated thymidine incorporation after 72 hours.

The immunosuppressive activities of IDO have been traditionally attributed to tryptophan starvation [9], which may cause generalized suppression of cellular proliferation. Under tryptophan-deficient conditions *in vitro*, human T cells stimulated non-specifically with mitogens enter the cell cycle and progress through the initial stages of G1 permitting IL-2 receptor upregulation and IL-2 synthesis, but undergo cell cycle arrest at a mid-G1 point [46]. Similarly, mouse T cells suffer cell cycle arrest and may become prone to apoptosis when deprived of tryptophan (33). We reasoned that in the absence of *in vivo* tryptophan catabolism by IDO, T_CD8_ may proliferate more vigorously in response to their cognate Ag. Therefore, we compared the expression levels of the classical proliferation marker Ki-67 among T Ag-specific T_CD8_ in WT and IDO^−/−^ mice. We found that a higher percentage of site IV-specific T_CD8_ expressed Ki-67 in IDO^−/−^ animals (Fig. 7A). This difference was less pronounced for site I-specific cells. The observed differences were not due to a global defect in the T cell compartment of IDO^−/−^ mice because WT and IDO^−/−^ splenocytes proliferated equally in response to several non-specific T cell mitogens, namely anti-CD3, PHA and ConA (Fig. 7B). Therefore, IDO appears to control the proliferative capacity of immunodominant T_CD8_, exemplified in this report by site IV in the T Ag system.

### IDO Controls Primary and Recall T_CD8_ Responses to Influenza A Virus (IAV) Immunodominant Epitopes

IDO has been studied extensively in the context of antitumor immunity, but its role in antiviral host defense is far less clear. Although T Ag is expressed as a result of SV40 transformation, it generally represents cell-associated tumor Ags. To extend our findings to antiviral T_CD8_, we compared T_CD8_ responses of B6 and IDO^−/−^ mice injected *i.p.* with the PR8 strain of influenza A virus (IAV). These responses are focused towards several PR8-derived peptides (Table 1), most notably towards NP_366_ and PA_224_, thus giving these two epitopes a “co-immunodominant” status within this well-characterized hierarchy [28], [30], [47]–[51].

**Figure 8:**
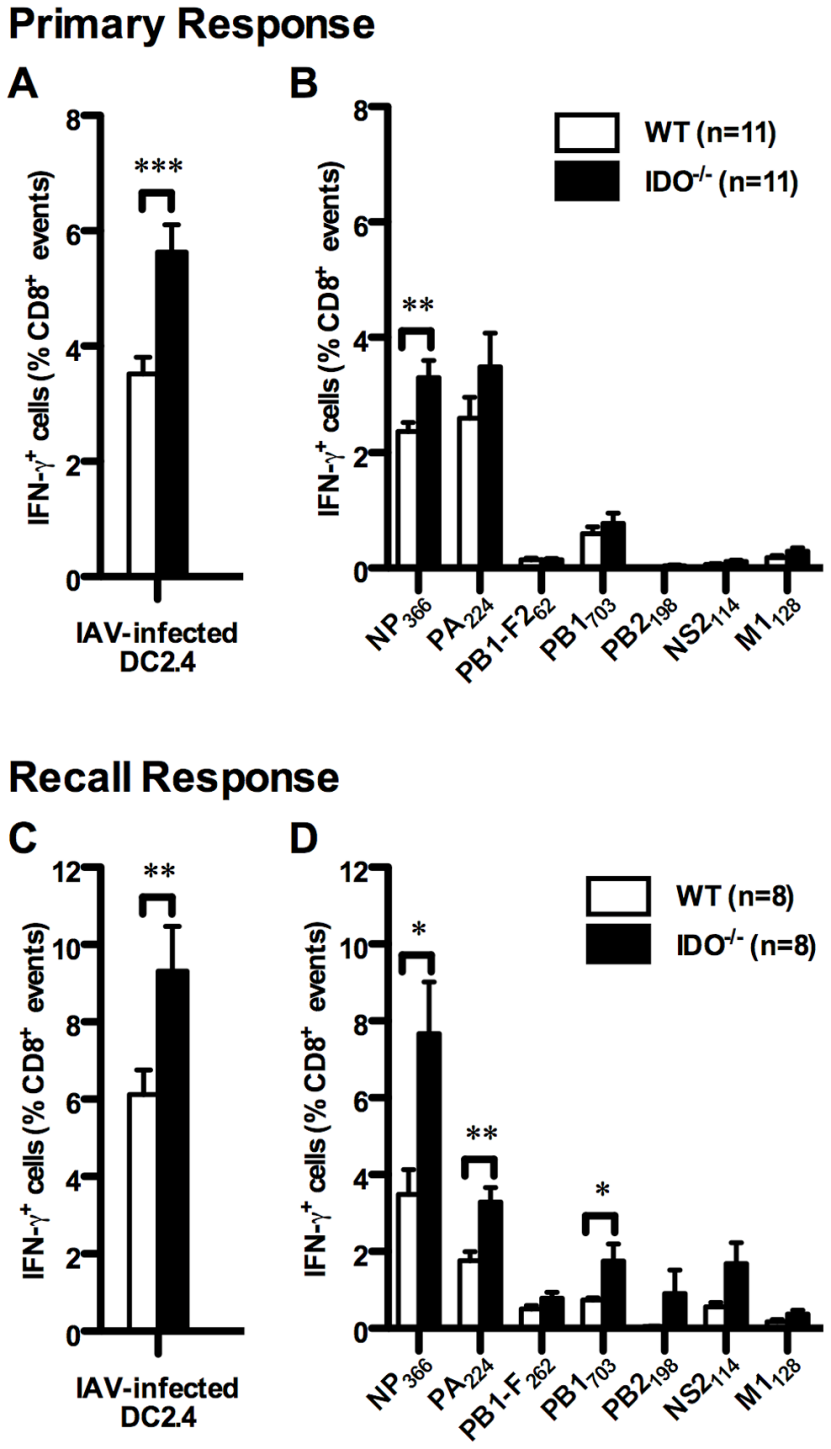
Primary and recall T_CD8_ responses of WT and IDO^−/−^ mice to influenza A virus. Mice were injected with the PR8 strain of IAV. Seven days later, splenocytes were prepared and restimulated *ex vivo* with IAV-infected DC2.4 cells (A) or with synthetic peptides corresponding to IAV epitopes (B). T_CD8_ responses were then quantified by ICS for IFN-γ. To assess recall responses, WT and IDO^−/−^ mice were primed with PR8 (H1N1) and boosted, one month later, with the X31 reassortant virus (H3N2). Seven days after the boost, the frequencies of total T_CD8_ synthesizing IFN-γ in response to IAV-infected DC2.4 cells (C) and those recognizing individual IAV-derived epitopes (D) were determined by ICS. Data are shown as mean IFN-γ^+^ cells as a percentage of CD8^+^events (± SEM) obtained from indicated numbers of mice per group, which were pooled from independent experiments.

IDO^−/−^ mice had a higher frequency of splenic T_CD8_ capable of producing IFN-γ after brief co-incubation with PR8-infected DC2.4 cells (Fig. 8A), which provides a rough estimate of the overall anti-IAV T_CD8_ response [8], [28]. Although the general rank order of IAV epitopes was maintained in IDO^−/−^ animals, lack of IDO enhanced the frequency of T_CD8_ specific for NP_366_ (Fig. 8B). In contrast, the relative frequencies of PA_224_-specific and subdominant T_CD8_ were similar between WT and IDO^−/−^ mice. The absolute numbers of NP_366_- and PA_224_-specific T_CD8_ were also elevated in IDO^−/−^ mice in comparison with WT controls (639,303±585 *vs.* 369,697±296 NP_366_-specific T_CD8_ per spleen; 712,658±1,269 *vs.* 429,570±695 PA_224_-specific T_CD8_ per spleen; p<0.001 and p = 0.05, respectively). In separate experiments, we found moderate increases in proportions and numbers of T_CD8_ recognizing NP_366_ after intranasal inoculation of PR8, which results in active respiratory infection (Fig. S7).

Given the importance of recall responses in protective immunity against viral pathogens, we asked whether IDO regulates the response intensity and immunodominance hierarchies of anti-IAV T_CD8_ when they re-encounter a similar virus. WT and IDO^−/−^ mice were primed with PR8 (H1N1) followed, 30 days later, by boosting with the X31 reassortant (H3N2) virus. PR8 and X31 share internal gene products, including nucleoprotein (NP) and acid polymerase (PA) that are targeted by T_CD8_. Importantly however, they express distinct and non-crossreactive hemagglutinin (H1 *vs*. H3) and neuraminidase (N1 *vs*. N2) glycoproteins on their surface [28], [30], [52], thus eliminating the complicating possibility of reduced viral doses due to antibody-mediated neutralization of the second inoculum in our prime-boost protocol. Seven days after boosting, we enumerated IAV-specific T_CD8_ by ICS for IFN-γ. As expected, secondary T_CD8_ responses to IAV were stronger than primary responses (Fig. 8C and Fig. 8D), and anti-NP_366_ T_CD8_ now dominated the recall response (Fig. 8D) as previously described [52]. The overall IAV-specific T_CD8_ response was more vigorous in IDO^−/−^ mice (Fig. 8C), and these animals mounted more robust responses against NP_366_ and PA_224_ when compared with WT controls (Fig. 8D). This was accompanied by an increase in absolute numbers of NP_366_- and PA_224_-specific T_CD8_ in IDO^−/−^ mice in comparison with WT controls (1,705,922±308,673 *vs.* 659,158±147,556 NP_366_-specific T_CD8_ per spleen; 733,886±101,066 *vs.* 328,955±56,931 PA_224_-specific T_CD8_ per spleen; p<0.01 for both). Finally, we noted an increase in the frequency of T_CD8_ recalling one of the subdominant IAV epitopes, namely PB1_703_, in IDO^−/−^ mice.

All together, our data demonstrate that systemic primary and secondary T_CD8_ responses to IAV are subject to IDO regulation. Also, as with the T Ag system, IDO functions to moderate immunodominance disparities in antiviral T_CD8_ responses.

## Discussion

In this investigation, we have examined the contribution of IDO to antitumor and antiviral T_CD8_ immunodominance. We have also addressed the role of IDO in regulation of cross-primed T_CD8_ responses to a clinically relevant tumor Ag, namely the SV40 large T Ag. This model is suitable for studying the relationship between oncogenic viruses, tumorigenesis and anticancer immunity. In fact, a causal relationship has recently been established between the human Merkel cell polyomavirus large T Ag and a rare but extremely aggressive type of cancer called Merkel cell carcinoma (MCC) [53]–[55]. Importantly, the presence of T Ag-specific T_CD8_ in MCC tumors is associated with better prognosis [53]–[57], and these cells are regarded as attractive candidates for adoptive transfer immunotherapy of MCC [57], [58]. Therefore, understanding the role of IDO in this model may provide clinically meaningful information on how antitumor T_CD8_ responses can be optimized.

Genetic deficiency of IDO or its pharmacological inhibition by 1-D-MT led to an increase in the T_CD8_ response to site IV, the most immunodominant peptide encoded by T Ag. Our work using the IAV infection/inoculation models yielded similar findings. Following *i.p.* inoculation of IDO^−/−^ mice with IAV, immunodominant NP_366_-specific T_CD8_ exhibited an amplified response in the periphery. Using this injection route, severe morbidity can be avoided since IAV is thought not to propagate within the peritoneal cavity to cause active infection [28]. This enabled us to examine the role of IDO in dictating antiviral T_CD8_ immunodominance without complications arising from an ongoing active infection. Furthermore, *i.p.* inoculation of IAV may be viewed as a model for flu vaccination in which killed (non-propagating) flu viruses are administered intramuscularly, which is also not the natural route for IAV infection. It needs to be emphasized that in our intranasal (*i.n.*) IAV infection model, which simulates active respiratory infection, IDO^−/−^ mice also exhibited a moderately increased T_CD8_ response to NP_366_. Finally, in the recall response of IDO^−/−^ mice to IAV, although NP_366_- and PA_224_-specific responses exhibited the most marked increases, a subdominant response (*i.e.*, PB1_703_) was also boosted, albeit to a lesser extent. Therefore, recall T_CD8_ responses to a broader range of IAV Ags can be augmented in the absence of IDO, which has clear implications for prime-boost immunization against IAV and potentially other rapidly mutating viruses.

Two very recent studies have addressed the role of IDO in immune responses to IAV in mice. Fox et al. reported that the bronchoalveolar lavage (BAL) fluid obtained from mice receiving 1-methyl-D,L-tryptophan in their drinking water and infected *i.n.* with the X31 strain of IAV contained more PA_224_-specific T_CD8_ [59]. In this study, control- and inhibitor-treated mice had similar frequencies of IAV-specific T_CD8_ in their mediastinal lymph nodes (MLNs). In contrast, Huang et al. found increased numbers of NP- and PA-specific T_CD8_ within the MLNs of X31-infected IDO^−/−^ mice compared with WT animals [24]. Our results are consistent with the findings of the latter investigation although one needs to keep in mind that we looked at splenic T_CD8_ that represent systemic responses as opposed to pulmonary T_CD8_ that provide antiviral protection locally. Huang et al. also found that priming with X31 protects WT and IDO^−/−^ mice against a subsequent challenge with a lethal dose of the PR8 strain. Interestingly, ablation of IDO failed to increase NP-, PA- and PB1-specific T_CD8_ numbers in the BAL and lungs of X31-primed mice that were challenged with PR8 [24]. In fact, NP_366_-specific T_CD8_ numbers were significantly reduced in the lung parenchyma of IDO^−/−^ mice. This is in contrast with our finding that in the absence of IDO, recall splenic T_CD8_ responses are augmented against the same Ags among a wide range of IAV-derived epitopes we tested (Fig. 8D). These inconsistencies likely stem from differences in the prime-boost protocols employed (*e.g.*, injection route, the order of IAV strains used sequentially for priming and boosting) and the systemic versus local nature of T_CD8_ responses examined. For instance, IAV infection is known to induce substantial IDO activity in the lungs but not in the spleen [23]. How local IDO expression and activity in various microenvironments may affect the nature, quality and breadth of antiviral T_CD8_ responses warrants further investigation.

In preliminary experiments, we have found no statistical difference between intranasally infected WT and IDO^−/−^ mice in terms of IAV viral particle levels in the lungs, which was judged by the relative abundance of matrix protein 2 (M2) (Fig. S8). Using a different readout (measuring 50% tissue culture infectious dose or TCID_50_ in MDCK plaque assays), Huang *et al* also reached a similar conclusion [24]. Therefore, it appears that the enhanced T_CD8_ response to IAV has no appreciable impact on viral clearance in the absence of IDO. Huang *et al* demonstrated that IDO^−/−^ mice lose less weight and recover more quickly from respiratory IAV infection [24]. It is important to note that IAV-inflicted morbidity and weight loss can be influenced by various host factors, especially by neutralizing antibodies to IAV.

Enhanced T cell responses to immunodominant epitopes may or may not translate to protective immunity [60]. In the case of the T Ag model for instance, the *in vivo* contribution of site IV-specific T_CD8_ and their therapeutic benefit is established in a mouse model of autochthonous brain cancer [61], [62]. In the case of certain viral infections, however, it is plausible to assume that narrowly focused T_CD8_ responses towards one or few epitopes may favor the emergence of pathogen escape mutants, especially in the case of rapidly mutating viruses such as IAV and human immunodeficiency virus (HIV), which may in turn compromise the overall effectiveness of T_CD8_-mediated immunity and protection [63], [64].

To address the role of IDO in regulation of T_CD8_ cross-priming, we immunized WT and IDO^−/−^ mice with allogeneic T Ag^+^ renal epithelial tumor cells (Fig. 2). These experiments revealed that IDO can negatively regulate spontaneously ongoing T Ag-specific and alloreactive T_CD8_ responses in the same host. Our finding that IDO suppresses T_CD8_ targeting MHC-mismatched kidney cells is in agreement with several previous reports indicating a similar role for IDO in inhibition of T_CD8_ responses to allogeneic murine fibrosarcoma cells [65], lungs [14] or pancreatic islets [66]. The latter study found that that adenovirus-based overexpression of IDO in mouse pancreatic islets attenuates the generation of central memory and effector memory cells among adoptively transferred alloreactive T_CD8_, whereas treatment with 1-MT promotes their generation. In the current work, we demonstrate for the first time that IDO can also suppress memory T_CD8_ responses in the context of IAV infection.

We previously demonstrated that nTreg cell depletion boosts immunodominant T_CD8_ in the T Ag and IAV inoculation models employed in this work [8]. On the other hand, there exists a functional link between nTreg cells and IDO in cancer. IDO expressed by plasmacytoid DCs (pDCs) present in tumor-draining lymph nodes can directly activate FoxP3^+^ Treg cells [16]. IDO^+^ pDCs can also prevent the conversion of Treg cells into proinflammatory TH17-like cells [67], which can be reversed in the presence of adoptively transferred Ag-specific T_CD8_. In mice with established B16 melanoma, oral administration of 1-D-MT and a lentivirus-based tumor vaccine led to conversion of many Treg cells to TH17-like cells within the tumor microenvironment, which was importantly associated with increased T_CD8_ activation [67]. In fact, it was recently demonstrated by the same group that such “reprogrammed” Treg cells exert helper activities to support T_CD8_ cross-priming in naive animals [68]. These observations prompted us to explore whether the absence of IDO in our model may affect nTreg cells numerically or functionally in the interest of a stronger T_CD8_ response to T Ag. Our findings indicate that nTreg cells are present in expected numbers in both naïve and T Ag-primed IDO^−/−^ mice and maintain their non-specific suppressive activity. More importantly, mAb-mediated pre-depletion or inactivation of nTreg cells further increased an already heightened response to site IV in IDO^−/−^ mice. This indicates that nTreg cells do not mediate the basal increase in these animals and that they act through a different mechanism to boost the immunodominant response in our model. It is possible that disrupting early IDO-Treg cross-talk that would lead to T_CD8_ activation requires robust participation by a high number of cognate T_CD8_ such as adoptively transferred OT-I or vaccine-primed T_CD8_ examined in the above studies by Sharma et al. This may not necessarily mirror the involvement of Ag-specific T_CD8_ present in low numbers in a pre-immune repertoire such as site IV-specific T_CD8_ in IDO^−/−^ mice prior to the inoculation of these animals with T Ag^+^ tumor cells. Nevertheless, based on our data, it is tempting to envisage clinical scenarios or vaccination strategies in which Treg cell depletion and IDO inhibitors can be combined to boost tumor- and virus-specific T cell responses in an additive fashion. Denileukin diftitox (*aka.* ONTAK), an engineered protein combining IL-2 and diphtheria toxin, has been used to deplete Treg cells and shown promise in boosting T_CD8_ in cancer patients [69], [70]. The IDO inhibitor 1-MT has been shown to be synergistic with several chemotherapeutic agents [40] and is currently used in clinical trials in patients with relapsed or refractory solid tumors [32], [71]. Therefore, combining Treg cell depletion with IDO inhibition may be a realistic therapeutic option for cancer.

Tryptophan catabolites are known to modulate certain aspects of immune responses. In this study, administration of L-Kyn to WT mice failed to inhibit the site IV-specific T_CD8_ response. This rules out the possibility that L-Kyn production mediates the suppressive effect of IDO in our system. In our preliminary experiments, we also found that treatment with L-Kyn similarly fails to diminish both immunodominant and subdominant splenic T_CD8_ responses to IAV (unpublished data). This is consistent with a previous report that non-toxic concentrations of L-Kyn do not inhibit the *in vitro* cytotoxic response of HLA-A0201-restricted human T_CD8_ response to an immunodominant peptide epitope of Epstein-Barr virus [72]. In the clinic, L-Kyn has been used as a prognostic factor, for instance in patients with diffuse large B-cell lymphoma receiving R-CHOP, an immunotherapeutic regimen consisting of rituximab, cyclophosphamide, hydroxydaunorubicin hydrochloride (doxorubicin), vincristine (Oncovin) and prednisolone [73]. A natural extension of our study and that of Weber et al. would be to correlate L-Kyn blood levels to memory T_CD8_ responses to common viral pathogens in similar settings.

In our initial experiments, we found that even though the frequency of site IV-specific IFN-γ^+^ T_CD8_ was increased in IDO^−/−^ mice (Fig. 1B), the mean fluorescence intensity of IFN-γ in WT and IDO^−/−^ T_CD8_ recognizing this epitope was comparable (Fig. S3). This suggested that the observed change may reflect a heightened proliferative capacity of these T_CD8_ in IDO^−/−^ mice without affecting other functions such as IFN-γ production on a per cell basis. It has been previously reported that IDO-expressing DCs activate a stress-response kinase called GCN2 kinase in T cells, thereby halting their proliferation [74]. We have found that when pulsed with T Ag-derived peptides, DCs generated from WT and IDO^−/−^ mice can both induce robust proliferation by splenocytes obtained from a previously immunized mouse. They also express comparable levels of the costimulatory molecules CD80 and CD86 as well as MHC class II (our unpublished data). Moreover, in our preliminary experiments, we have not detected any noticeable difference in intracellular expression of C/EBP-homologous protein (CHOP), a downstream marker of GCN2 activation [74], between WT and IDO^−/−^ T Ag-specific T_CD8_. Nevertheless, site IV-specific T_CD8_ exhibited a high expression level of Ki-67 indicating that they had proliferated more vigorously in IDO^−/−^ animals. This was not due to a global change in the proliferative capacity of IDO^−/−^ T cells since several T cell mitogens triggered similar responses by WT and IDO^−/−^ splenocytes. Therefore, collectively, lack of IDO is associated with an increase in immunodominant Ag-specific T_CD8_ proliferation *in vivo*. We propose that immunodominant T_CD8_ clones may be more prone to tryptophan starvation. They may proliferate more rapidly than other clones once they encounter peptide:MHC I complexes they are meant to recognize. By the same token, in the absence of IDO, immunodominant T_CD8_ may expand even more rapidly early in the course of the immune response, which may reinforce their ability to “dominate” subdominant T_CD8_.

Finally, C57SV and KD2SV cells, the two T Ag^+^ cell lines used in this study, express IDO, which cannot be enhanced by treatment with IFN-γ (Fig. S9), a pro-inflammatory cytokine and a potent inducer of IDO [75], [76]. It will be interesting to compare the magnitude, quality and breadth of T Ag-specific T_CD8_ responses in WT mice inoculated with IDO-sufficient and IDO-deficient versions of T Ag^+^ cells. Such experiments will help determine whether/how tumor-derived IDO may influence tumor-specific T_CD8_ and their immunodominance hierarchies.

In summary, in this work, we have demonstrated for the first time that: i) IDO suppresses the cross-primed T_CD8_ response to an immunodominant tumor Ag; ii) IDO-mediated suppression of immunodominant T_CD8_ is Treg cell- and L-Kyn-independent, and is accompanied by a higher *in vivo* proliferative capacity of these clones; iii) IDO regulates systemic primary and recall T_CD8_ responses to influenza. Future work will address the exact biochemical mechanism(s) underlying the observed effect and test the efficacy of IDO inhibitors when combined with other immunotherapeutic modalities including but not limited to Treg cell inactivation.

## Supporting Information

Figure S1 **Gating strategy for detection of T Ag-specific T_CD8_ by ICS for IFN-γ.**
(TIF)

Figure S2 **The absolute numbers of bulk, T Ag-specific T_CD8_ (A) and site IV-specific T_CD8_ (B) appear to be moderately increased in IDO^−/−^ mice although statistical significance was not reached.**
(TIF)

Figure S3 **T Ag-specific T_CD8_ from primed WT and IDO^−/−^ mice exhibit comparable IFN-γ MFI levels.**
(TIF)

Figure S4 **T Ag-primed WT and IDO^−/−^ mice harbor comparable quantities of tetramer^+^ site I- and site IV-specific T_CD8_ in their spleen.**
(TIF)

Figure S5 **Naïve and T Ag-primed WT and IDO^−/−^ mice have similar frequencies and absolute numbers of CD4^+^CD25^+^ cells in their spleen.**
(TIF)

Figure S6 **Treatment with an anti-CD25 mAb (clone PC61) reduces the frequencies and absolute numbers of splenic nTreg cells in WT and IDO^−/−^ mice.**
(TIF)

Figure S7 **The absolute number of immunodominant NP_366_-specific T_CD8_ appears to be moderately increased in intranasally flu-infected IDO^−/−^ mice although statistical significance was not reached.**
(TIF)

Figure S8 **Intranasally infected WT and IDO^−/−^ mice have comparable levels of the flu matrix 2 protein (M2) in their lungs.**
(TIF)

Figure S9 **IDO is detectable in C57SV and KD2SV cell lines but cannot be upregulated by IFN-γ treatment.**
(TIF)

Table S1 **Frequencies of T Ag-specific, IFN-γ^+^ T_CD8_ in the spleen of IDO^+/+^ and IDO^−/−^ mice inoculated with C57SV fibrosarcoma cells.**
(TIF)

Table S2 **Frequencies and absolute numbers of T Ag-specific, IFN-γ^+^ T_CD8_ in the spleen of IDO^+/+^ and IDO^−/−^ mice inoculated with KD2SV kidney epithelial tumor cells.**
(TIF)

Table S3 **Frequencies and absolute numbers of T Ag-specific, IFN-γ^+^ T_CD8_ in the spleen of 1-D-MT- and vehicle-treated B6 wild-type mice inoculated with C57SV fibrosarcoma cells.**
(TIF)

Table S4 **Frequencies and absolute numbers of T Ag-specific, IFN-γ^+^ T_CD8_ in the spleen of L-kynurenine- and vehicle-treated B6 wild-type mice inoculated with C57SV fibrosarcoma cells.**
(TIF)
