# Supplementary figures and images for: Suppression of Immunodominant Antitumor and Antiviral CD8+ T Cell Responses by Indoleamine 2,3-Dioxygenase
Source: PLoS One. 2014 Feb 28;9(2):e90439. doi: 10.1371/journal.pone.0090439 (PMC3938761; doi:10.1371/journal.pone.0090439)

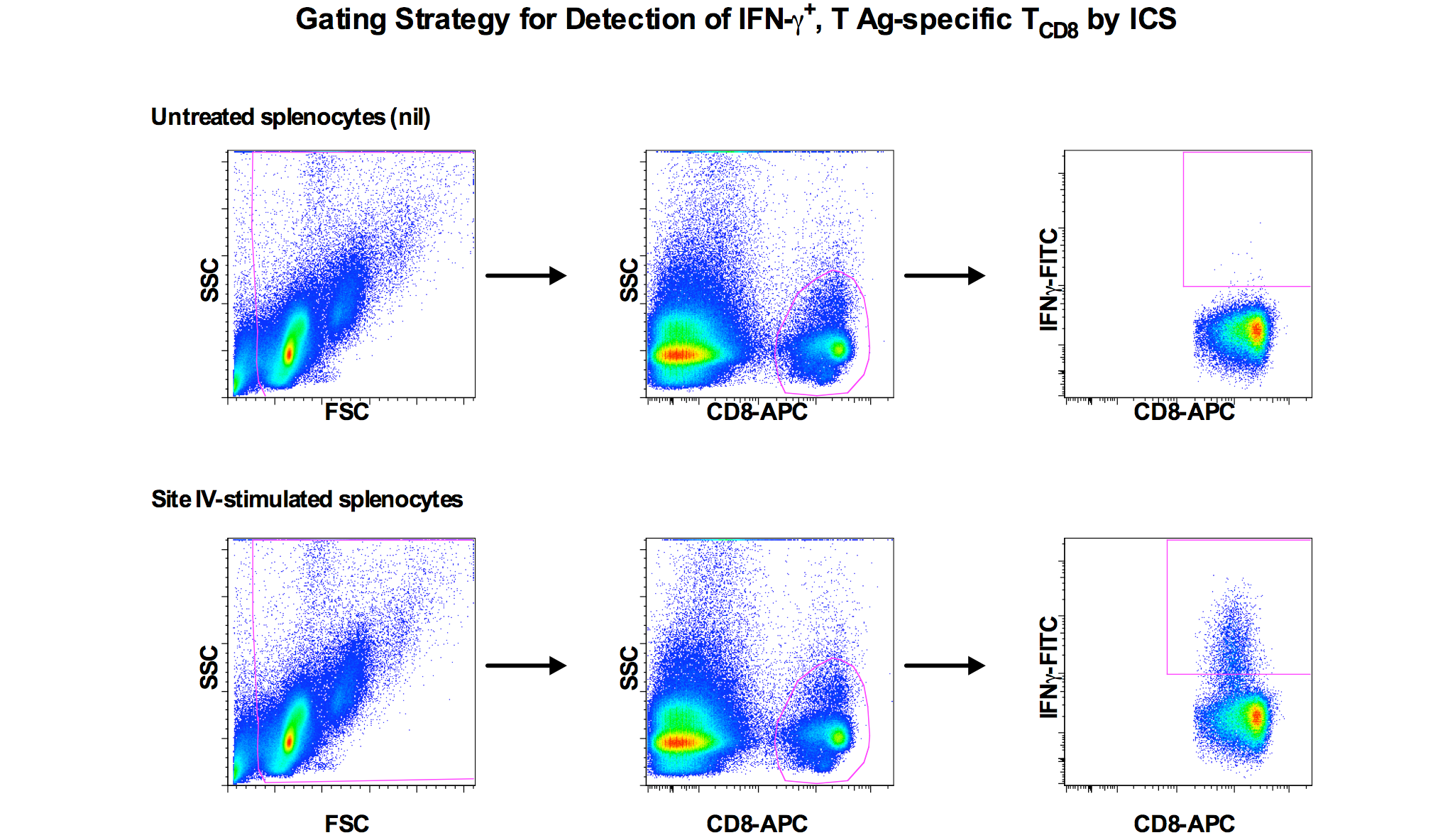

Supplement: Figure S1 — Gating strategy for detection of T Ag-specific TCD8 by ICS for IFN-γ. (TIF) [file pone.0090439.s001.tif]

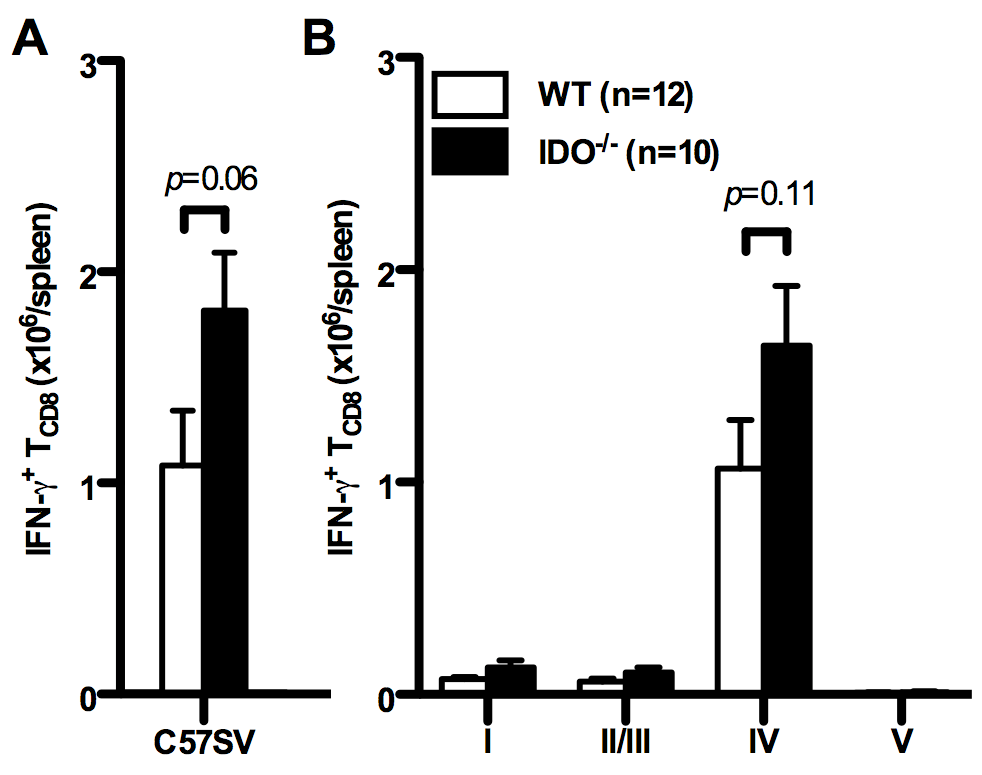

Supplement: Figure S2 — The absolute numbers of bulk, T Ag-specific TCD8 (A) and site IV-specific TCD8 (B) appear to be moderately increased in IDO−/− mice although statistical significance was not reached. (TIF) [file pone.0090439.s002.tif]

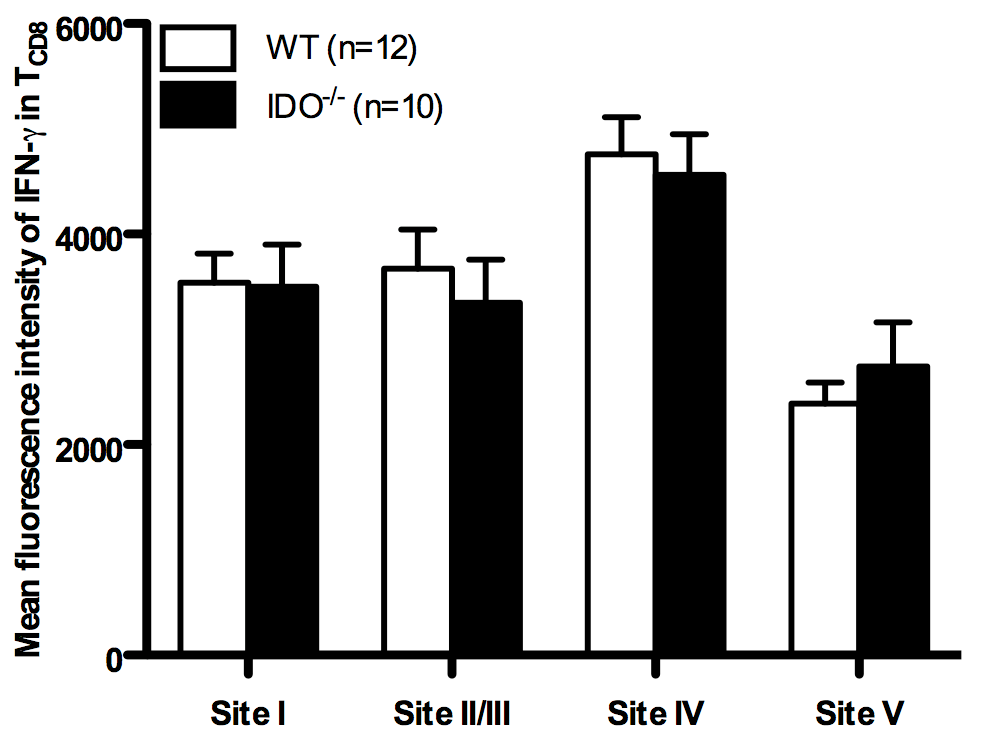

Supplement: Figure S3 — T Ag-specific TCD8 from primed WT and IDO−/− mice exhibit comparable IFN-γ MFI levels. (TIF) [file pone.0090439.s003.tif]

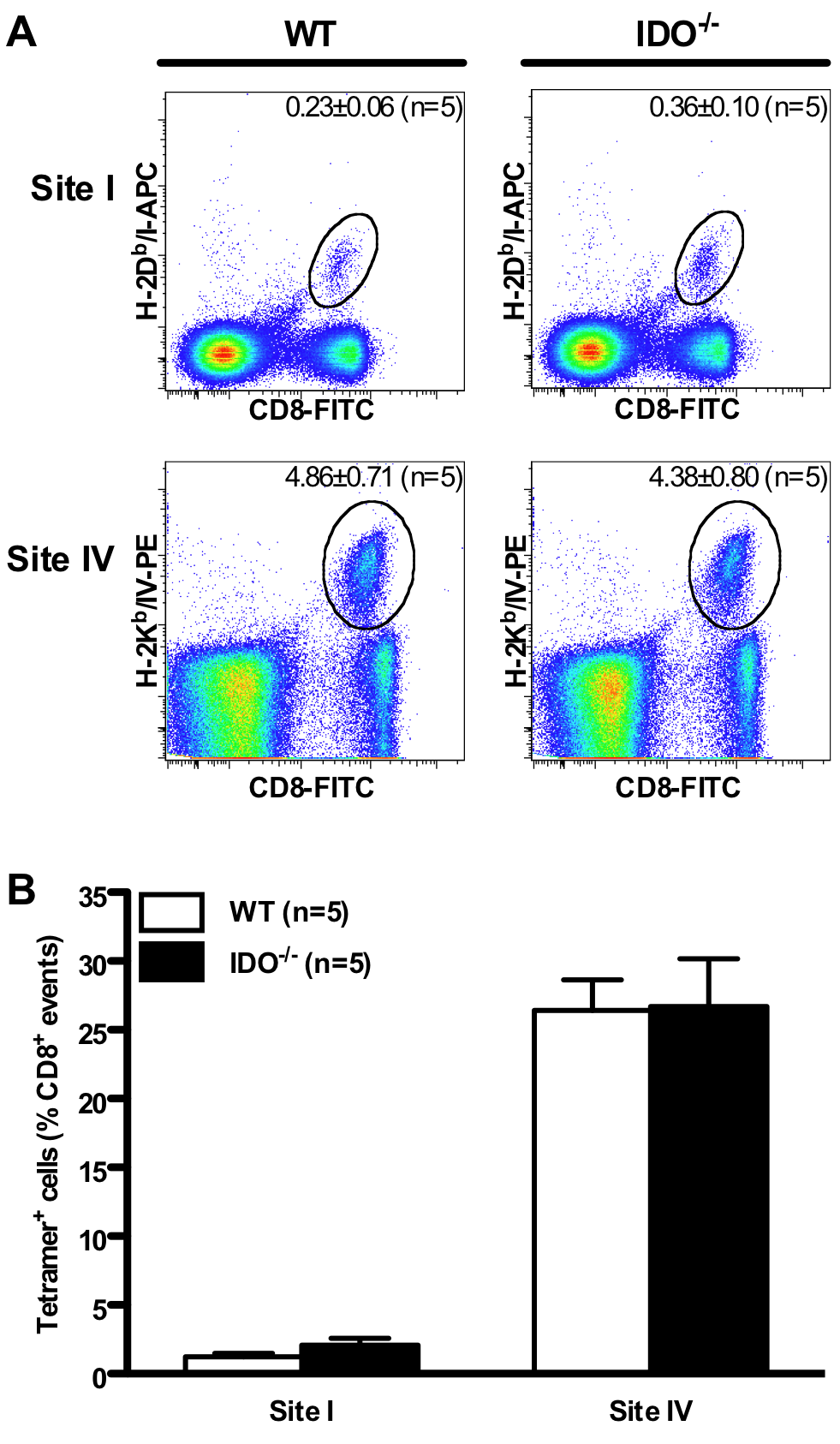

Supplement: Figure S4 — T Ag-primed WT and IDO−/− mice harbor comparable quantities of tetramer+ site I- and site IV-specific TCD8 in their spleen. (TIF) [file pone.0090439.s004.tif]

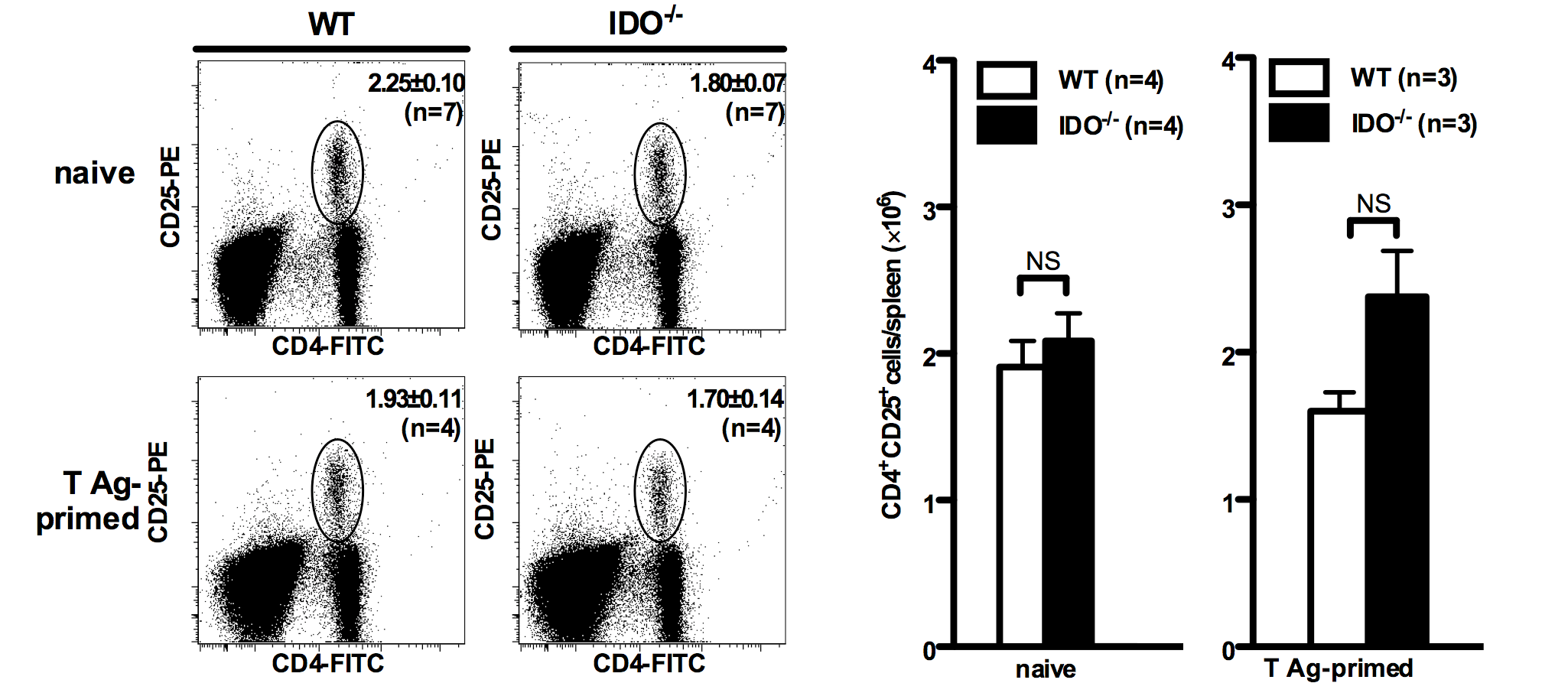

Supplement: Figure S5 — Naïve and T Ag-primed WT and IDO−/− mice have similar frequencies and absolute numbers of CD4+CD25+ cells in their spleen. (TIF) [file pone.0090439.s005.tif]

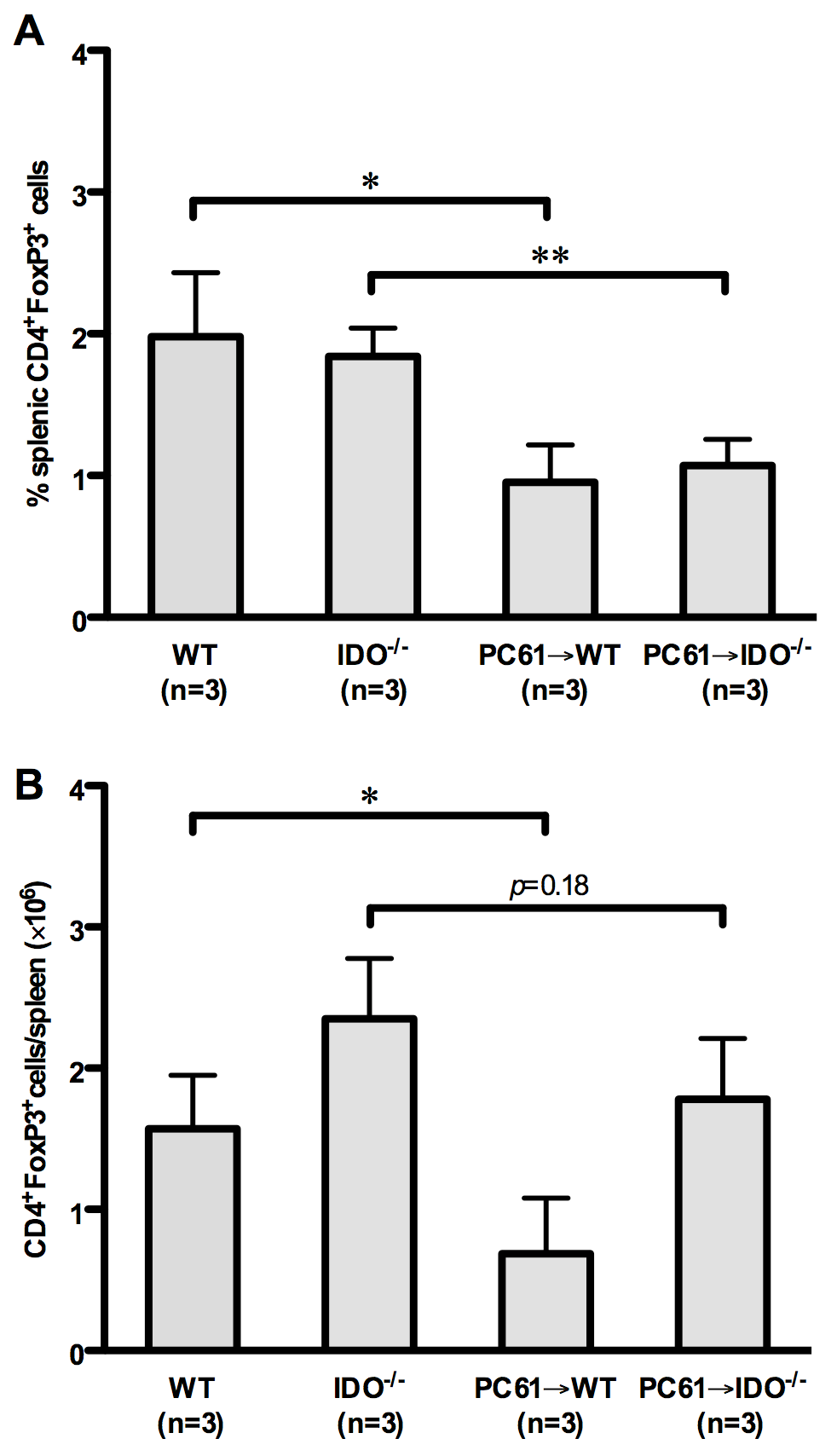

Supplement: Figure S6 — Treatment with an anti-CD25 mAb (clone PC61) reduces the frequencies and absolute numbers of splenic nTreg cells in WT and IDO−/− mice. (TIF) [file pone.0090439.s006.tif]

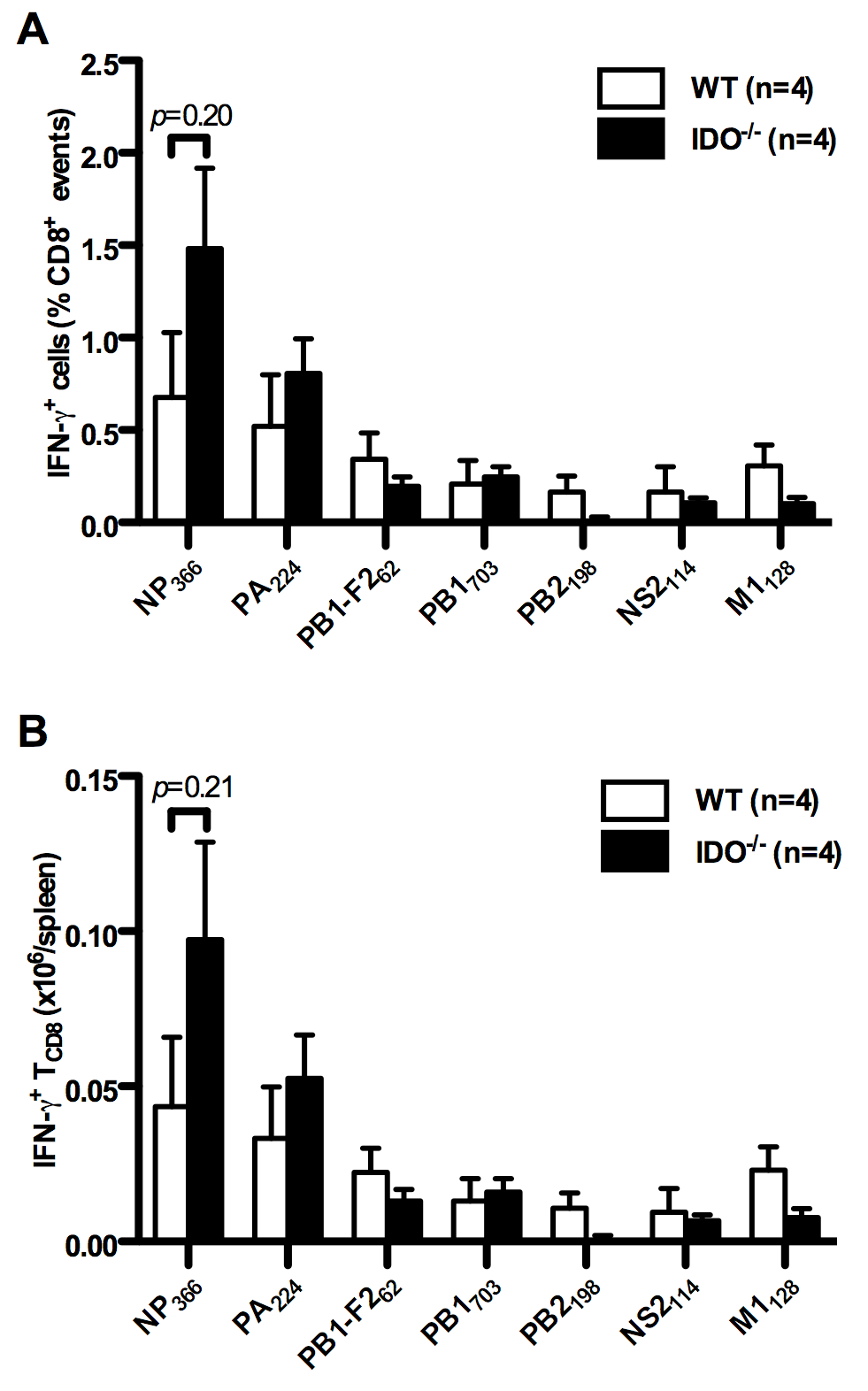

Supplement: Figure S7 — The absolute number of immunodominant NP366-specific TCD8 appears to be moderately increased in intranasally flu-infected IDO−/− mice although statistical significance was not reached. (TIF) [file pone.0090439.s007.tif]

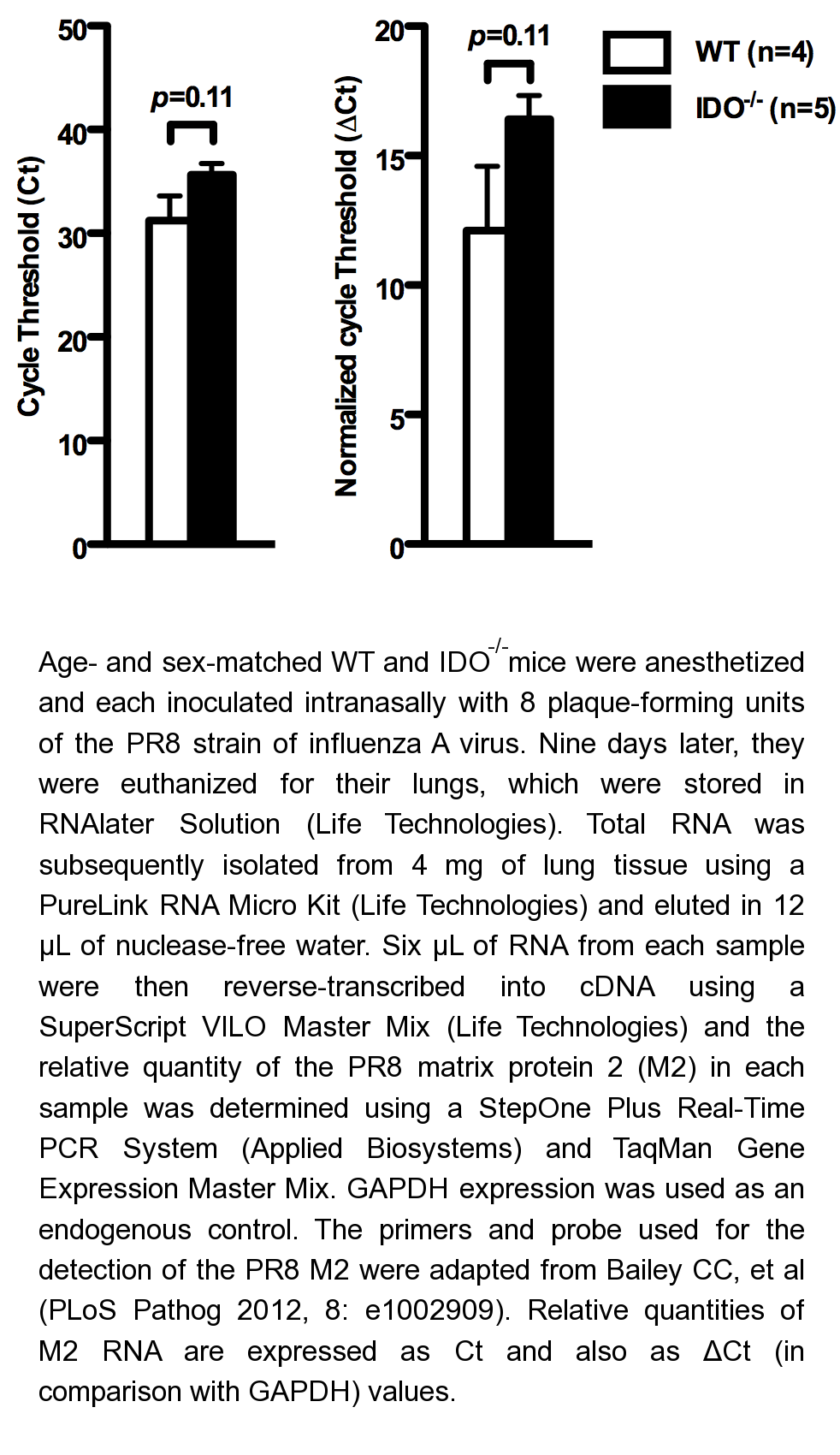

Supplement: Figure S8 — Intranasally infected WT and IDO−/− mice have comparable levels of the flu matrix 2 protein (M2) in their lungs. (TIF) [file pone.0090439.s008.tif]

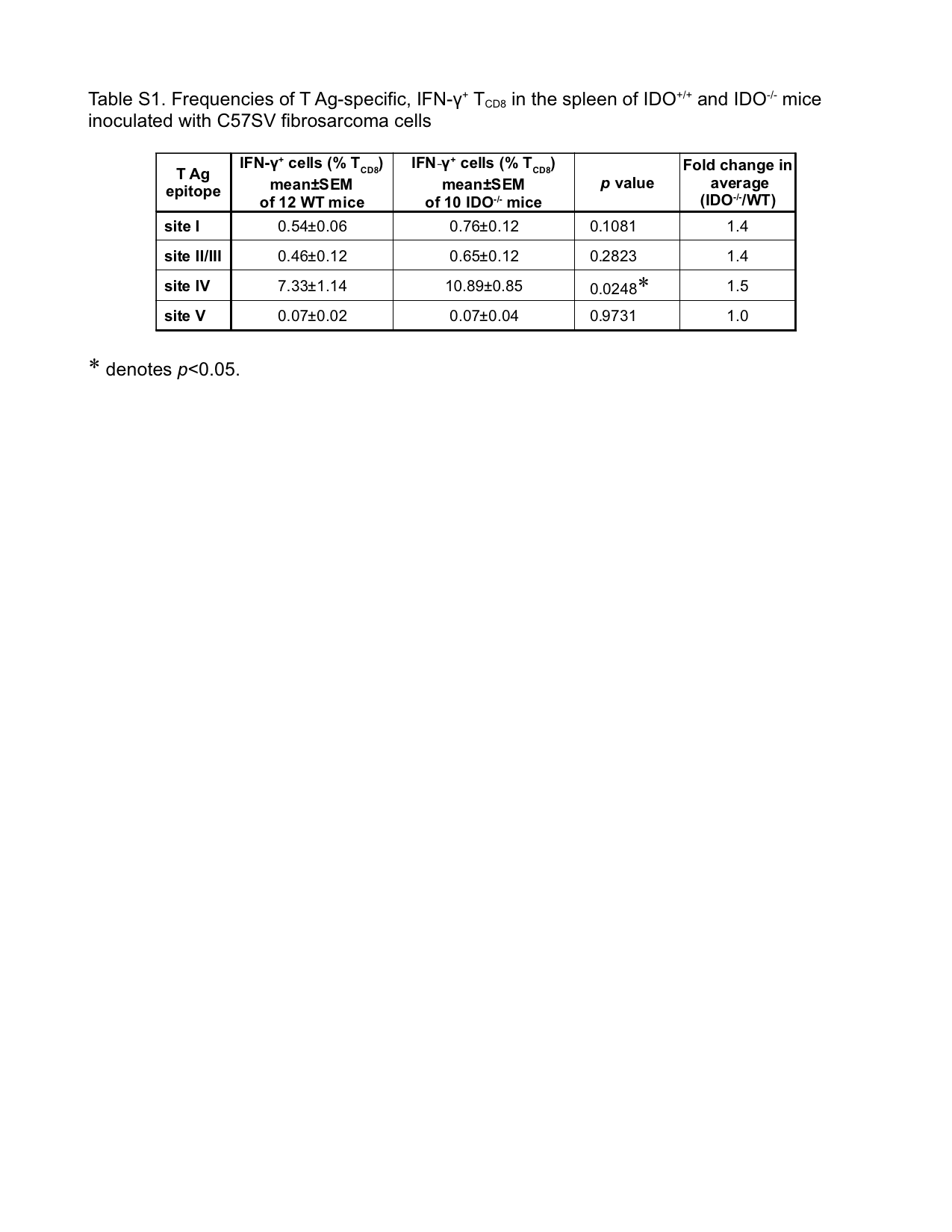

Supplement: Table S1 — Frequencies of T Ag-specific, IFN-γ+ TCD8 in the spleen of IDO+/+ and IDO−/− mice inoculated with C57SV fibrosarcoma cells. (TIF) [file pone.0090439.s010.tif]

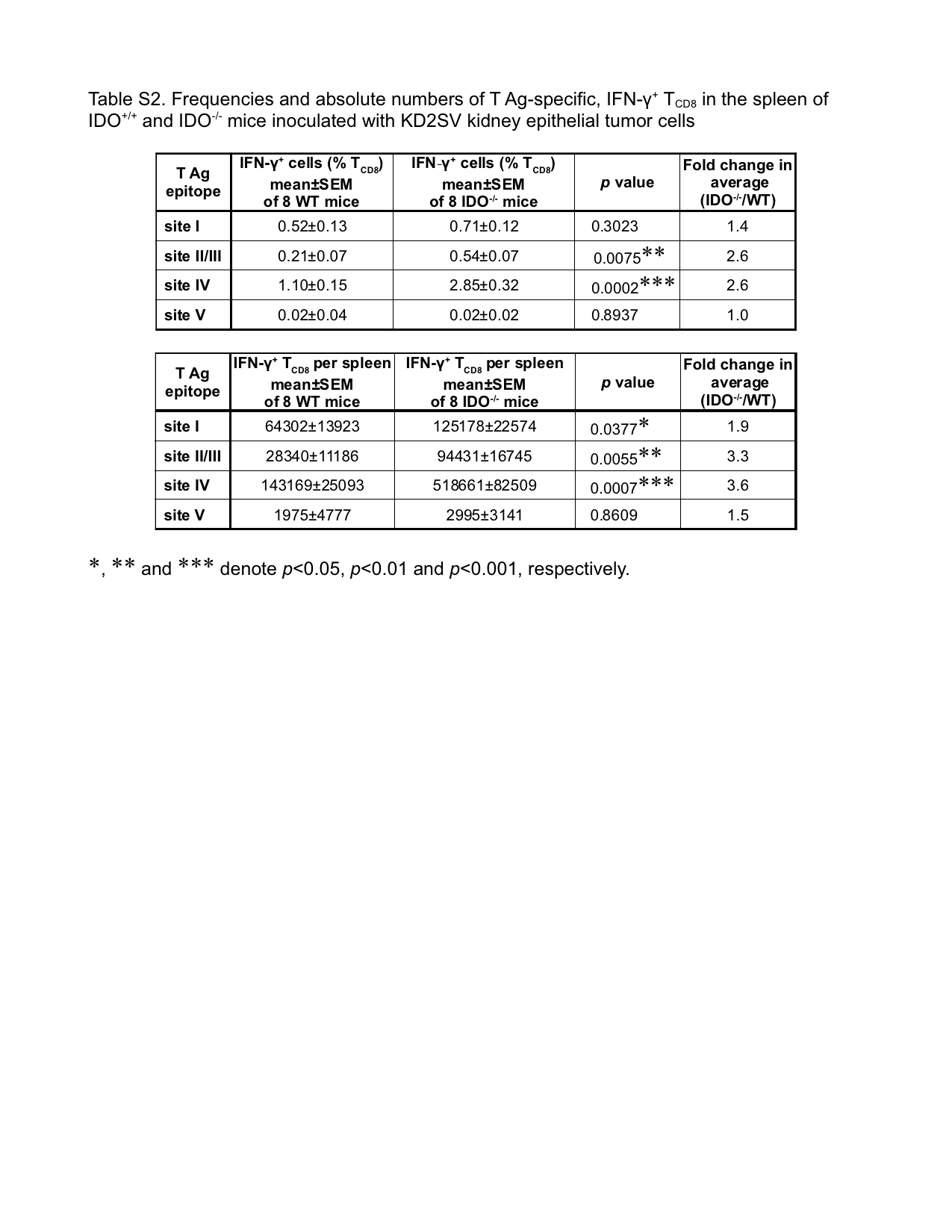

Supplement: Table S2 — Frequencies and absolute numbers of T Ag-specific, IFN-γ+ TCD8 in the spleen of IDO+/+ and IDO−/− mice inoculated with KD2SV kidney epithelial tumor cells. (TIF) [file pone.0090439.s011.tif]

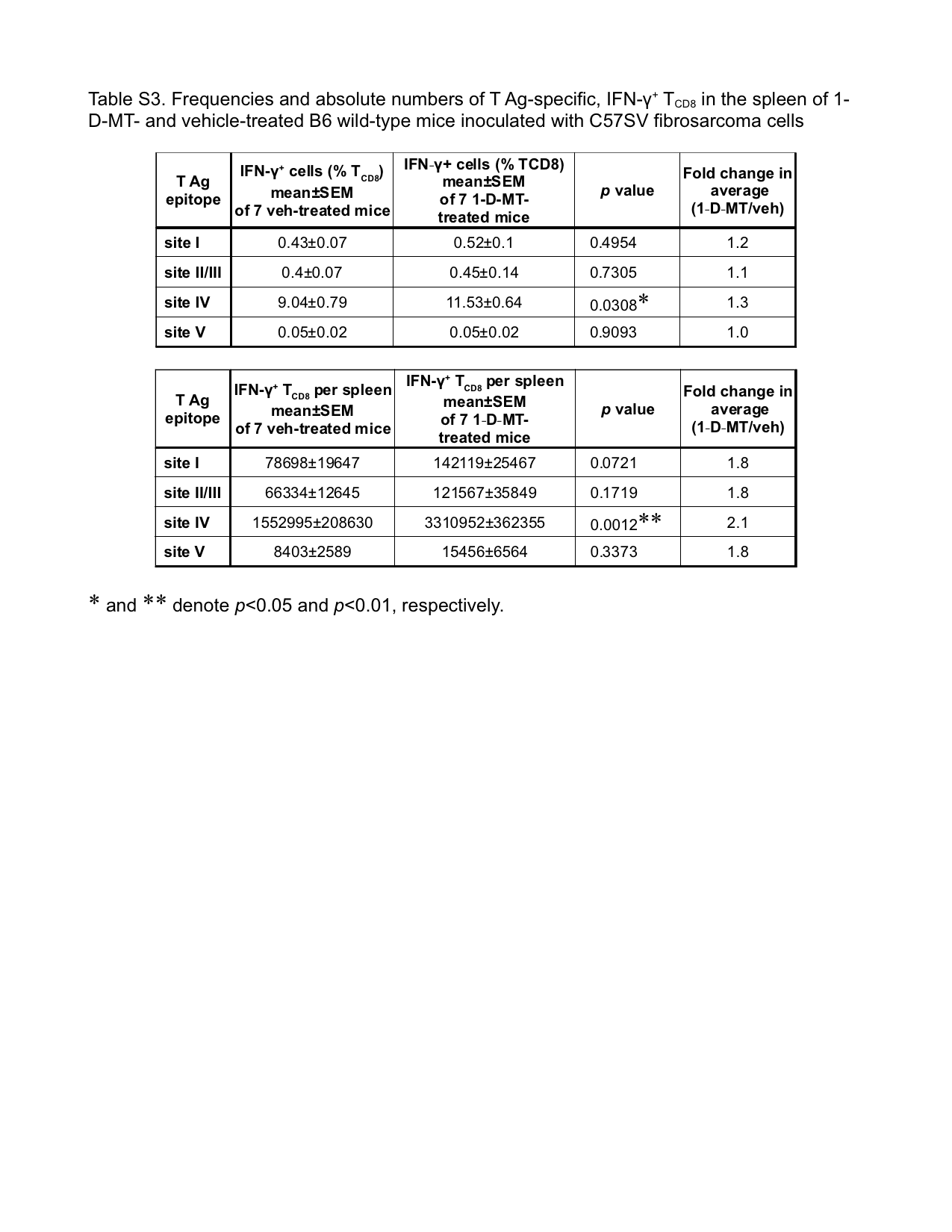

Supplement: Table S3 — Frequencies and absolute numbers of T Ag-specific, IFN-γ+ TCD8 in the spleen of 1-D-MT- and vehicle-treated B6 wild-type mice inoculated with C57SV fibrosarcoma cells. (TIF) [file pone.0090439.s012.tif]

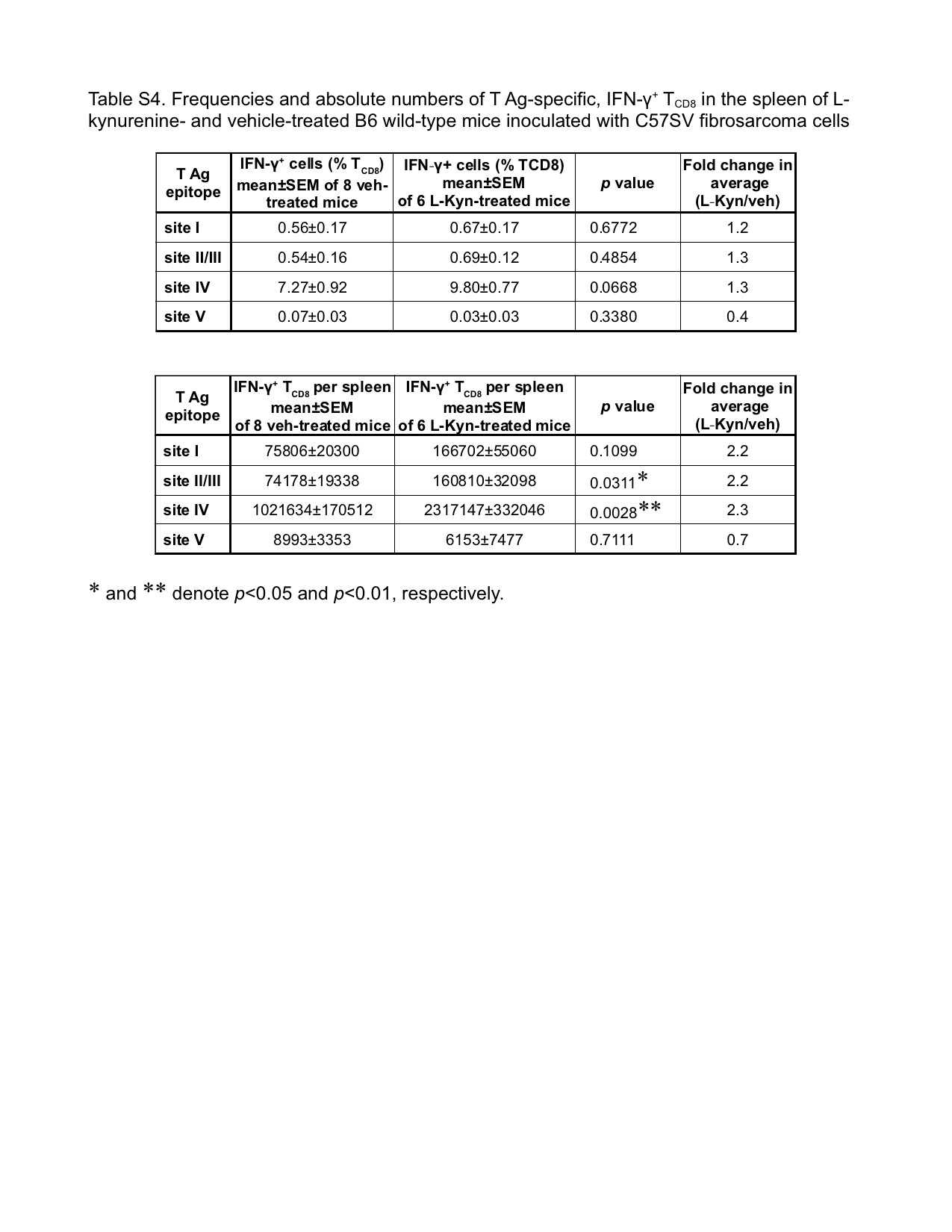

Supplement: Table S4 — Frequencies and absolute numbers of T Ag-specific, IFN-γ+ TCD8 in the spleen of L-kynurenine- and vehicle-treated B6 wild-type mice inoculated with C57SV fibrosarcoma cells. (TIF) [file pone.0090439.s013.tif]
